# Towards better environmental performance of wastewater sludge treatment using endpoint approach in LCA methodology

**DOI:** 10.1016/j.heliyon.2017.e00268

**Published:** 2017-03-17

**Authors:** Isam Alyaseri, Jianpeng Zhou

**Affiliations:** a Department of Civil Engineering, Al-Muthanna University, Samawah 72001, Iraq; b Department of Civil Engineering, Southern Illinois University Edwardsville, IL, USA 62026-1800

**Keywords:** Environmental science

## Abstract

The aim of this study is to use the life cycle assessment method to measure the environmental performance of the sludge incineration process in a wastewater treatment plant and to propose an alternative that can reduce the environmental impact. To show the damages caused by the treatment processes, the study aimed to use an endpoint approach in evaluating impacts on human health, ecosystem quality, and resources due to the processes. A case study was taken at Bissell Point Wastewater Treatment Plant in Saint Louis, Missouri, U.S. The plant-specific data along with literature data from technical publications were used to build an inventory, and then analyzed the environmental burdens from sludge handling unit in the year 2011. The impact assessment method chosen was ReCipe 2008. The existing scenario (dewatering-multiple hearth incineration-ash to landfill) was evaluated and three alternative scenarios (fluid bed incineration and anaerobic digestion with and without land application) with energy recovery from heat or biogas were proposed and analyzed to find the one with the least environmental impact. The existing scenario shows that the most significant impacts are related to depletion in resources and damage to human health. These impacts mainly came from the operation phase (electricity and fuel consumption and emissions related to combustion). Alternatives showed better performance than the existing scenario. Using ReCipe endpoint methodology, and among the three alternatives tested, the anaerobic digestion had the best overall environmental performance. It is recommended to convert to fluid bed incineration if the concerns were more about human health or to anaerobic digestion if the concerns were more about depletion in resources. The endpoint approach may simplify the outcomes of this study as follows: if the plant is converted to fluid bed incineration, it could prevent an average of 43.2 DALYs in human life, save 0.059 species in the area from extinction, and make a 62% reduction in the plant’s current expenses needed by future generations to extract resources per year. At the same time it may prevent 36.1 DALYs in humans, save 0.157 species, and make a 101% reduction in current expenses on resources per year, if converting to anaerobic digestion.

## 1 Introduction

The growing of the world's population and the improvement in standards of living across the world causes higher demand for efficient treatment of wastewater. This demand can be met either by using more efficient treatment processes in wastewater treatment plants (WWTPs) or establishing new plants. The need for improvements in quality and increasing quantities of treated water adds more burdens to the environment due to the need to construct and operate more facilities. The goal of the treatment in WWTPs went beyond only protecting surface or ground water. The new generations of WWTPs have to integrate the concerns of energy efficiency, carbon footprint and other sustainability issues with the concerns of water quality (e.g. Hospido et al., 2004; Papa et al., 2013; Bertanza et al., 2015; Abuşoglu et al., 2016). A wastewater treatment plant is considered efficient if it is capable of harvesting the potential energy available in the organic matters and nutrients found in the wastewater (Lazarova et al., 2012). The sludge treatment unit in the plant is a place where the organic matters and nutrients can be processed for energy and nutrients recovery. Johansson et al. (2008) showed that it is important for the environmental outcome of sludge treatment options to minimize the direct gaseous emissions and recover nutrients. Hong et al. (2009) showed that the global warming potential generated from incineration and melting processes can be significantly reduced when the heat is used for electricity generation.

However, there are many options for wastewater and sludge treatment available. Each option or treatment train will need comprehensive assessment to evaluate the overall environmental impacts. Such assessment can be achieved through the use of life cycle assessment (LCA) methodology (Tillman et al., 1998).

LCA studies use a life cycle impact assessment (LCIA) method to describe the impacts on the environment (Bishop, 2000). There are two approaches used by LCIA methods; midpoint and endpoint. The midpoint approach adopts the categories that lay in the middle of the cause-effect chain without going further to calculate their final damages such as the damages on human health or ecosystem. Some examples of these midpoint categories are global warming that is measured in kg CO_2_ eq., acidification measured in H^+^ moles eq., and ozone layer depletion measured in kg CFC-11 eq (the trichlorofluoromethane or the freon-11).

The endpoint approach goes further by changing the midpoint impact categories to more specific damage categories on humans and ecosystem. For example, the endpoint approach changed the amount of carcinogens calculated in the classification step into equivalent cancer cases in humans measured as disability adjusted life years (DALY). For a given process or product, it can calculate the fraction of species affected by the nutrients introduced to environment or the surplus energy or expenses needed by future generations to extract the resources they need due to the current use of resources. Several methods are now using the endpoint approach (such as Eco-indicator 99, IMPACT 2002, and ReCipe) (Goedkoop et al., 2010; Jolliet et al., 2003; Humbert et al., 2014; Goedkoop et al., 2013).

The endpoint approach has higher uncertainty than the midpoint approach due to the need for more assumptions, data, and calculation steps to perform a complete environmental model (Goedkoop et al., 2013). However, it is helpful for decision makers, designers, and manufacturers to see the end impacts caused by the decisions they made, processes they designed, or products they produced. The endpoint approach makes it easier for them to understand and evaluate the final impacts of their selections. It is also useful for the regulatory agencies to use the endpoint method to determine the final impacts of the regulations and explain them to society (Bare et al., 2000). The damage categories in the endpoint approach correspond to areas of protection that represent the basis for decisions in sustainable development or policy making (Goedkoop et al., 2013).

The international workshop held in Brighton (England) on May 2000, suggested that “methodologies should be as transparent as possible whilst still providing the desired level of accuracy”, and for the complex models, there has to be a sufficient consensus within the scientific community about the documentations used (Bare et al., 2000). The process of changing a unit of substance released from a process into equivalent cancer cases in human or disappearance for some species in environment, on the endpoint level, is long and needs to facilitate a more structured and informed weighting process than the midpoint level. Besides the transparency difficulty, data availability represents one of the biggest constraints that are limiting the use of the endpoint approach. However, with the increasing interest in LCA methodology and the increase in the number of epidemiological and environmental studies (Zamagni et al., 2012), the link between the inventory and potential end damages on human, ecosystem, and resources for most categories becomes established (Goedkoop et al., 2013).

Although the midpoint methods are easier to perform (less data and assumptions required), they have complications in evaluating the impacts. It is hard for the decision makers to understand the impact of the classified inventory (e.g. C_6_H_6_ eq., 2, 4-D eq., or CFC-11 eq.) on our life, or what is the difference between releasing one kg or one ton of a substance in this or that location (Bare et al., 2000). For this reason, the damages in the endpoint methods such as the ReCipe methodology were aggregated into three endpoint categories: human health, ecosystem quality, and depletion in resources. These categories are easier for many decision-makers to evaluate than the midpoint impact categories which are considered vague by some studies (Kirk et al., 2005). A midpoint approach may also face the problem of the improper selection of the relevant impact categories especially when dealing with wide system boundary cases. For example, a life cycle assessment practitioner may focus on a certain midpoint category, while high impact may come from another. Svanström et al. (2005) showed that the best sludge handling option from an environmental point of view is related to the selected impact category in the life cycle impact assessment methodology. In the endpoint approach, the practitioner can focus on final damages which usually are a combination of all midpoint impact categories. Also, when there are multiple categories, the endpoint approach is considered to be the most valuable if aggregation was desired (Bare et al., 2000).

The use of the endpoint approach in LCA studies is still limited. Among 35 sewage sludge LCA studies reviewed by Yoshida et al. (2013) only six studies used the endpoint LCIA method. From those, three of the studies used the endpoint method alone, while the rest used only the midpoint categories in the method for comparison with other LCIA methodologies.

Major drivers in sludge LCA studies are usually related to energy or phosphorous recovery and alternatives (conventional or innovative) comparison (Yoshida et al., 2013). Most of the midpoint approach studies were conducted in plants with specific impact categories in the minds of decision makers. The use of the midpoint approach in such studies would be adequate. But, when a plant (such as the one in this case study) has no specific criteria for comparison among several alternatives and wants to enhance the environmental performance of processes, the endpoint approach would be more appropriate for the evaluation of overall environmental performance of these alternatives. However, the trade-off between the two approaches depends on the goal and scope of the study and the ability of the decision makers to interpret the results (Goedkoop et al., 2010).

Many studies focused on one or multiple specific midpoint impact categories, but did not consider evaluating the endpoint damages on human health or ecosystem quality (e.g. Suh and Rousseaux, 2002; Hospido et al., 2005; Johansson et al., 2008; Lederer and Rechberger, 2010). As an example, Tarantini et al. (2007) focused on air acidification, eutrophication, green house effects, resource depletion and photo-oxidant formation on the midpoint level, while Hong et al. (2009) used four categories for comparison between multiple treatment trains also in the midpoint level. Limited studies describe the endpoint damages from wastewater treatment (Svanström et al., 2014; Tomei et al., 2016; Bertanza et al., 2015). There is still a need for more studies that describe the endpoint damages or the overall impacts of wastewater or sludge treatment due to variation in spatial and temporal conditions on each case study and along with the difference in the concerns from one place to another.

Also, the studies on wastewater treatment and sludge management can be classified into two types based on their final scope. The first type tries to analyze the environmental performance of the existing processes (e.g. Hospido et al., 2004), while the second tries to compare the environmental performance of different options or treatment trains (e.g. Murray et al., 2008; Pasqualino et al., 2009; Suh and Rousseaux, 2002). The combination of the two scopes is essential for better understanding of problems and solutions related to every case study. The scope of this study will focus on both.

## 2 Methodology

### 2.1 Objective of the study

The aim of the research described in this paper is to use the endpoint approach in LCA methodology to evaluate the environmental performance of the existing sludge treatment train used in Bissell Point Wastewater Treatment Plant (BPWWTP). Also the research aim to test number of alternatives and to provide set of recommendations to decision makers for better environmental performance associated with the treatment processes based on an endpoint approach.

### 2.1 Case study description

BPWWTP is a 567.8 × 10^3^ m^3^/day (150 million gallon/day) design plant located in St. Louis, Missouri. The design population equivalent is 1.5 × 10^6^. This study was conducted to facilitate the decision makers’ efforts to analyze the existing and available options considering environmental concerns. The plant receives combined stormwater and municipal wastewater from the St. Louis community. Secondary sludge resulting from the trickling filter process is pumped back and mixed with the primary sludge, then pumped to the sludge holding wells for temporary storage. From there, the sludge is sent to a scum concentrator which removes floating debris at the surface of the sludge. Traveling out of the scum concentrator, the sludge is injected with polymer and pumped to the belt filter presses (BFPs). The sludge cake that comes out of the BFPs moves onto a conveyor belt and is deposited into an equalization basin with screws that convey the cake to one side of the basin. From there, it falls down through a chute to the multiple hearths incinerators. The bottom ash resulting from incineration is dropped down from the incinerator and mixed with plant effluent water to form ash slurry. The ash slurry is pumped to two lagoons. Once the lagoons are filled, the ash is transported via trucks to a landfill that is approximately 10 kilometers away. Reject water from BFPs and drain from wet scrubber are taken back to the primary clarifiers. The diagram in the Supplementary Materials (LCI for Wastewater Sludge Treatment in Bissell Point WWTP) shows the processes flow.

### 2.2 Alternative scenarios

Three alternatives were proposed; the use of fluid bed incineration followed by landfilling ashes, the use of anaerobic digestion followed by digestate’s landfilling, and anaerobic digestion followed by land farming application.

The first scenario was proposed by facility engineers and included energy recovery to offset the energy consumption for incineration. Fluid bed incineration becomes more attractive in terms of capital and operation costs (Fytili and Zabaniotou, 2008). According to Dangtran et al. (2000), it is more economical to install a new fluid bed incinerator than to rehabilitate an existing multiple hearth incinerator. The advantages of a fluidized bed incinerator are lower auxiliary energy requirement, higher capacity, shorter residence time, and lower emissions (Kown, 1978; Dangtran et al., 2000). The benefit of fluidization is to achieve ideal mixing between the sludge and the combustion air. The most noticeable impact of the better burning atmosphere provided by a fluidized bed incinerator is seen in the limited amount of excess air required for complete combustion of the sludge. Using LCA methodology to compare six wastewater sludge treatment scenarios, Houillon and Jolliet. (2005) showed that incineration in fluid bed and agricultural spreading have the lowest non-renewable primary energy consumption. Other than the use of fluid bed incineration and utilizing the heat into power generation, the process in this scenario is the same as the existing scenario.

The second alternative scenario is to use anaerobic digestion with biogas recovery for power generation. In this process, the wastewater sludge is thickened in a gravity thickener then digested by anaerobic bacteria in anaerobic digesters so the sludge is stabilized to a safer and more easily dewatered substance. Digestate will be dried in lagoons prior to being finally disposed of in a landfill. Effluent from gravity thickeners in this scenario is taken back to the primary clarifiers. Wide applications of anaerobic digestion are motivated by its energy-related benefit (formation of biogas that has high calorific value which can be used to produce heat and electricity) (Cao and Pawlowski, 2012). Murray et al. (2008) conducted a study to compare nine scenarios for sludge treatment, and found that anaerobic digestion is generally the optimal treatment technology. Murray et al. study showed that the results of the life cycle impact assessment are highly correlated with the energy demand of the treatment technology and the overall ranking of the different options closely matches the technology’s rank in operation energy. Pasqualino et al. (2009) proposed using biogas for electricity and heat generation to reduce the environmental impacts from wastewater treatment processes. Hong et al. (2009) showed that the global warming potential generated from incineration and melting processes can be significantly reduced when the heat is used for electricity generation.

One method of disposing of digested sludge is to apply it to agricultural land to avoid using fertilizer. The land application of sludge has become one of the common practices (Hall, 1995; Wang et al., 2008; etc.). But, one limitation of applying sludge to land is the various toxins, especially heavy metals, which can be transferred to soil. These heavy metals can harm the soil plant system and may further pose threat to human health (Turkdogan et al., 2003; Wang et al., 2003 and 2008). Hence, the application of digested sludge for land has to consider the accumulation of heavy metals in land. To test the environmental benefits/burdens of such final disposal method, another scenario of anaerobic digestion followed by land application was suggested. The distance to the nearest farms in the area is around 41.7 km (25 miles). The process includes sending the digested sludge to the existing lagoons and when dried, it will be taken to farms for application.

### 2.3 System boundaries

The careful selection of system boundaries has a big influence on LCA (Lundin et al., 2000). In this case study, secondary sludge is returned to the primary clarifiers and the mixed sludge is taken from there. The boundaries of the system start at the point where the sludge is taken from the primary clarifiers. The reference flow for assessment was defined as one kg of mixed sludge in dry basis (1 Dry kg) with 70% volatile solids.

The system boundaries include the phases of construction and operations. Construction materials include production, manufacturing and end-of-life phases. To normalize to the reference flow, service life for all mechanical equipment was assumed to have an average of 40 years, and the service life for building materials was 70 years. Minor consumable materials (such as offices supplies, monitoring devices, etc.) and labor were not included in this study. The contribution of office buildings and capital goods that are used for all scenarios were neglected and it’s commonly accepted to ignore the contribution of such elements in life cycle inventory (Tarantini et al., 2007).

The useful products that may be produced from the proposed alternatives such as biogas or heat were included. The transportation (fuel and the machinery) to deliver the end product to the point of end disposal was included. The system boundary includes the fly ash emissions, the bottom ash residual, and the digested sludge. Land occupied by processes was included. Maintenance was not included due to the lack of data.

The model included the sludge conditioning system, polymer used, and treatment of the washwater. The water mixed with the polymer was neglected. The dust and noise from combustion were not included. The process of land application was assumed to include the heavy metals emissions to the soil.

### 2.4 Inventory analysis

A life cycle inventory (LCI) was performed by collecting inventory data from the case study (BPWWTP) and can be seen in the Supplementary Materials (LCI for Wastewater Sludge Treatment in Bissell Point WWTP). The daily information provided from the sludge treatment unit in the plant was; flow rate, power and natural gas consumption, total press sludge flow, total polymer used for dewatering, and total dry sludge incinerated. In the year 2011 (selected as an average year), the actual flow rate to the plant was an average of 520.5 × 10^3^ m^3^/day (137.5 million gallon/day), and the dry sludge incinerated was an average of 105.5 ton/day. Construction materials were measured onsite. Several publications were used to compensate for missing data from the plant and to build the inventory for the alternative scenarios proposed. The different data sources are specified in the Supplementary Materials.

For the purpose of contribution analysis, the inventories were classified into eight groups (emissions, construction materials, electricity, ash to landfill, transportation, WWTP burdens, chemicals, and natural gas). WWTP burdens include the burdens from the sewer grid, pump station, and the recycled wastewater from dewatering (the washwater) process. Chemicals only included the polymer used for dewatering.

The amount of electricity used by the sludge handling processes in the plant was estimated by the plant's engineers to be 18% to 22% of the total electricity consumed in the plant. The consumption in the sludge handling unit was then calculated (equal to an average of 0.353 Kwh/dry kg). The natural gas consumption was 3.49 MJ/dry kg. For fly ash treatment, the technology used in the existing multiple hearths incinerator is a venturi scrubber/impingement tray scrubber. Regulated emissions (CO, NO_x_, VOC, PM_10_, SO_2_, PM_2.5_ and Pb) were taken from the EPA National Emission Inventory. Data for other emissions were taken from Ecoinvent database or from technical publications like Doka (2006) or USEPA (1995). Tables A1 through A8 show the inventory of the existing and proposed scenarios.

### 2.5 Life cycle impact assessment method

According to Lazarova et al. (2012), CML, Eco-indicator 99, and Eco-points 97 are the most commonly used LCIA methods in the LCA water sector. The CML method is a midpoint approach that does not support damage assessment and weighting into a single score result. Eco-point 97 (the new version of this method is called Ecological Scarcity) can calculate impacts in a single score but it uses 30 individual impact categories (e.g. NO_x_, SO_x_, and Pb_(air)_), and does not show the final damages to human health and ecosystem or depletion of resources. Eco-indicator 99 is one example of the methods that can interpret the inventory into an endpoint damages on human health and ecosystem quality and to estimate the overall impacts in a single score. The method can calculate the contribution of every inventory element in a single score which helps in defining the major contributors for further sensitivity or uncertainty analysis. According to Corominas et al. (2013), the method was used by three LCA studies in the wastewater treatment sector.

ReCipe is the new version of Eco-indicator 99 method and CML 2002 method. The method combines midpoint and endpoint methodologies in a consistent way. In this method, inventory data are classified into 17 impact categories. Endpoint assessment is obtained by multiplying the amount of emissions released under every category by a damage factor (measured in DALY, Species.yr, or $). Procedures to obtain damage factors are explained in (Goedkoop et al., 2013). Impact categories in this method are the climate change, ozone depletion, terrestrial acidification, freshwater eutrophication, marine eutrophication, human toxicity, photochemical oxidant formation, particulate matter formation, terrestrial ecotoxicity, freshwater ecotoxicity, marine ecotoxicity, ionising radiation, agricultural land occupation, urban land occupation, natural land transformation, mineral resource depletion, and fossil fuel depletion. These impact categories are then grouped in three damage categories (human health, ecosystem quality and resources depletion), which are further divided by normalization factors and weighted into a single score (points, Pt or millipoints, mPt). A typical product in LCA shows a much smaller environmental damage than the normalization values. So most LCA results are in micro points or even in nano points if uses a scaling factor of 1, for which a scaling factor of 1000 are used and the result in millipoints. The version “World ReCipe H/A” will be used in this study which refers to the normalization values of the world with the average weighting set based on the Hierarchist perspective. Uncertainty analysis was performed based on Monte Carlo Simulation. Model uncertainty or uncertainty from LCIA method was not covered in this research.

## 3 Results and discussion

### 3.1 Existing treatment analysis

**Fig. 1:**
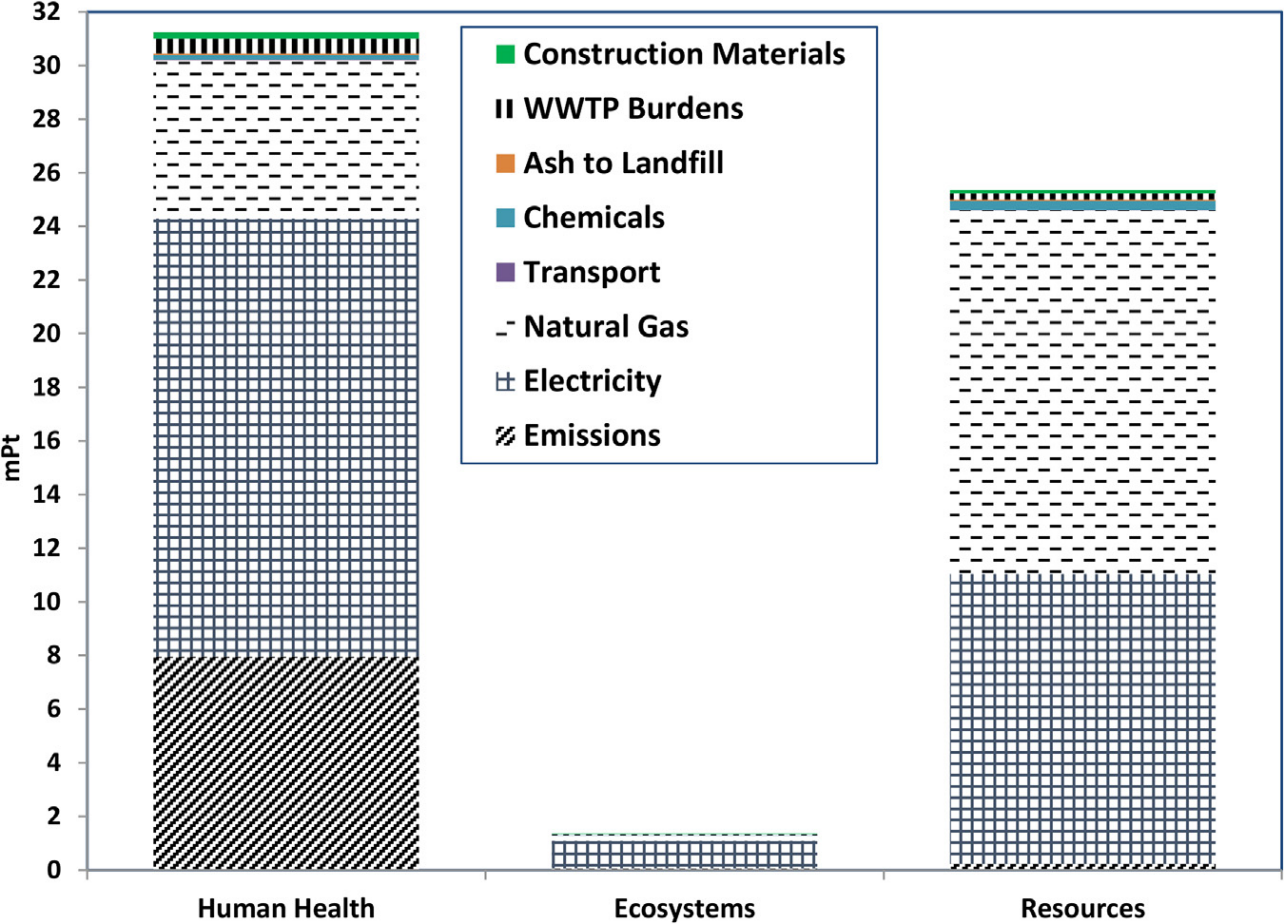
Damage Assessment Analysis for Endpoint Damage Categories of the Existing Multiple Hearths Incineration Process in Bissell Point WWTP for 1 kg Dry Solids Using ReCipe Endpoint Method (H) V1.05/World ReCipe H/A/Weighting.

Fig. 1 shows the contribution of the groups of inventories to each one of the damage categories. The Figure shows higher damages on human health and resources than on ecosystem. The Figure shows that significant impacts on human health are coming from electricity, emissions, and natural gas (16.3 mPt, 7.96 mPt, and 5.91 mPt, respectively). The damages to resources mainly come from natural gas and electricity consumed in the plant (13.6 mPt, and 10.8 mPt, respectively). Also, electricity is the most significant contributor to the damage on ecosystem. These show the need for an energy conservation and emission reduction in any proposed alternative.

As for impact categories, the major impact from processes was related to fossil depletion ($2.83 equal to 25.4 mPt per each dry kg) (Table 1). The second highest impact is related to climate change affects human health (4.75 × 10^−7^ DALY equal to 14.1 mPt per each dry kg), while the third highest impact is on particulate matter formation (3.65 × 10^−7^ DALY equal to 10.7 mPt per each dry kg) which is mainly related to emissions from incineration plus the emissions from electricity and natural gas production. As expected, there was no significant contribution to respiratory from organics because most organics are burned during incineration. The impact on ozone layer depletion, fresh water, terrestrial and marine ecotoxicity, and ionizing radiation was not significant due to small amounts of emissions related to these categories.

**Fig. 2:**
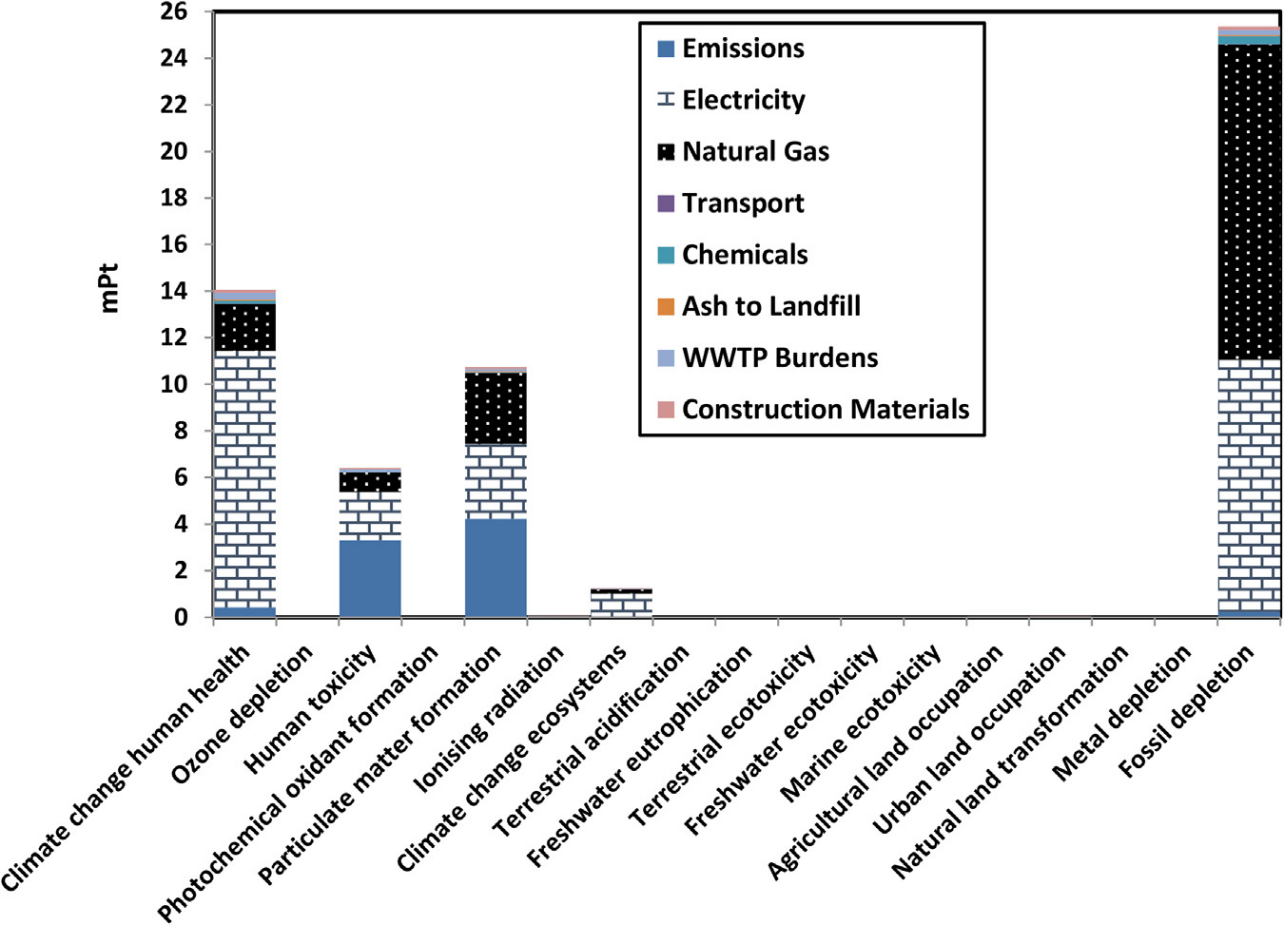
Analysis of Impact Categories of the Existing Multiple Hearths Incineration Process in Bissell Point WWTP for 1 kg Dry Solids Using ReCipe Endpoint Method (H) V1.05/World ReCipe H/A/Weighting.

Fig. 2 shows that the emissions are the major contributor to the human toxicity and particulate matter formation, and shows how important to improve the efficiency of fly ash treatment in the plant. The most common impact category considered by sewage sludge management studies reviewed by Yoshida et al. (2013) was the global warming potential (measured in kg CO_2_ eq.), while Corominas et al. (2013) showed that among 45 studies reviewed on wastewater treatment, 38 of them considered the global warming potential and none of them analyzed the particulate matter formation or human toxicity potentials. Employing weighting of ReCipe Method in this case study shows the need to pay equal attention to these categories, or the need to use a damage category (e.g. human health) which is a combination of these impact categories. Fig. 2 shows that both particulate matter formation and carcinogens have more impacts than the emissions related to climate change (only 14.1 mPt of single environmental scores of impacts resulted from processes related to the climate change category vs. 17.1 mPt to particulate matter formation and human toxicity).

Fig. 2 also shows the impacts of each group of inventory to the process on 17 impact categories. The Figure shows that the environmental impacts of the operation phase of sludge treatment processes are significantly greater than the impacts of the construction phase. This result is consistent with results from other studies that neglected the environmental impacts related to the construction of facilities (e.g. Murray et al., 2008; Hospido et al., 2004; Hong et al., 2009). Suh and Rousseaux (2002) neglected the environmental impacts related to minor consumable materials and the construction materials of the facilities because the impacts are negligible when compared to those of the long operation period (more than 30 years). Fig. 2 shows a small contribution from chemicals used. The plant used relatively small amounts of polymer compared to other plants (1.51 kg/dry ton in this case study vs. 5 kg/dry ton by Suh and Rousseaux (2002), 7.1 kg/dry ton by Houillon and Jolliet (2005), and 5.5 kg/dry ton by Hospido et al., (2005)). The short distance to the landfill (9.6 km) is the reason for the low impacts from transportation. This distance is shorter than the distances assumed by other studies (60 km in Lederer and Rechberger, 2010 and 25 km in Hospido et al., 2004). Fig. 2 shows that among all contributors in this model, the emissions from the incineration process, natural gas, and electricity used for operations have the most significant contribution and they are dominating the life cycle phases. The attention has to focus on these contributors to reduce the related impacts from the process.

The total sludge incinerated in the BPWWTP in the year 2011 was 38283 tons. The annual damages can be calculated using the ReCipe method, which supports the analysis of environmental burdens from the treatment processes in terms of the final damages to human health, eco-system, and resources. For example, the annual damage to human health was an average of 40.2 DALYs. The annual surplus expenses needed by future generations to extract the same resources currently consumed in the plant were an average of $1.07 × 10^8^.

### 3.2 Comparative analysis

**Fig. 3:**
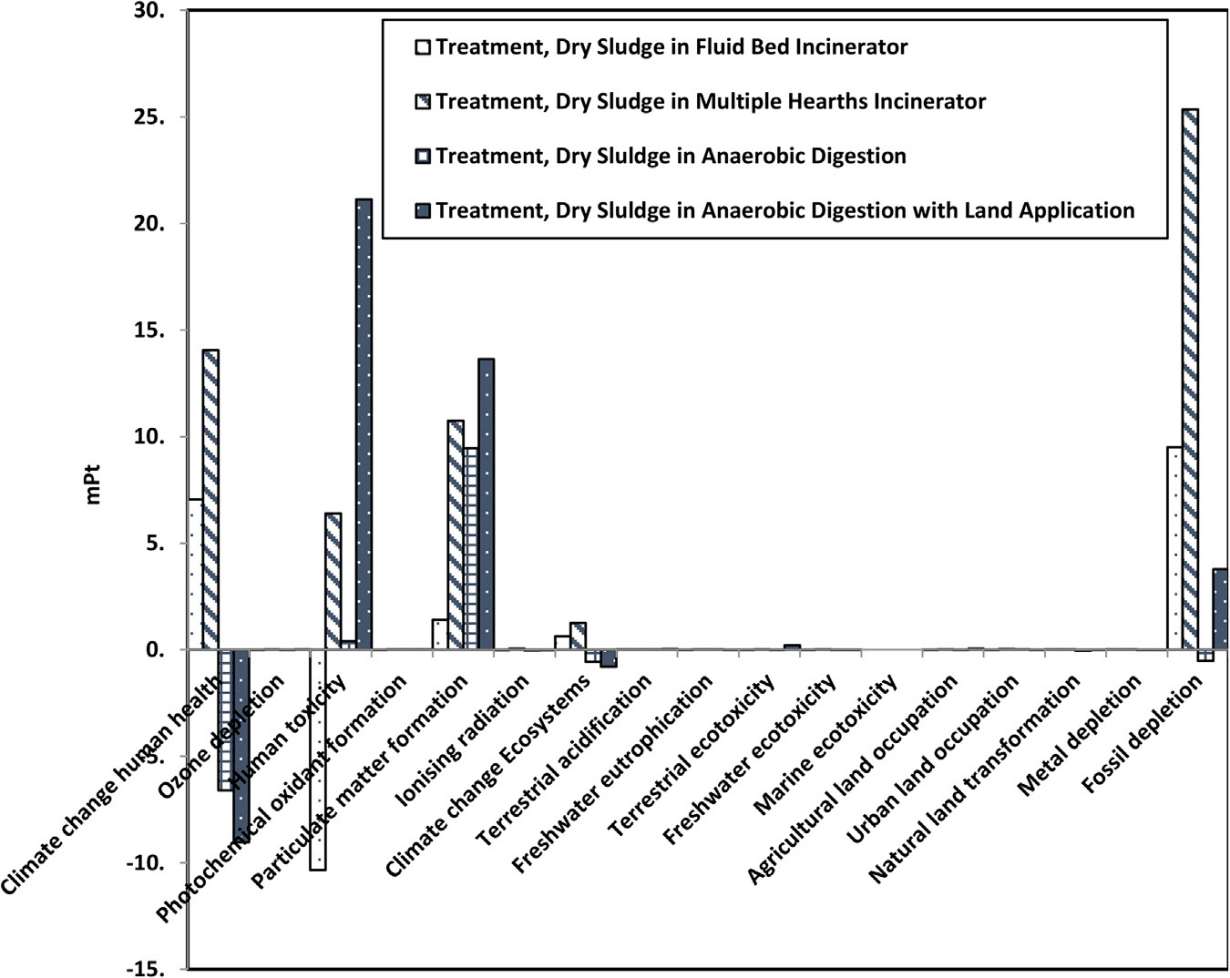
Comparison of Impact Categories of the Existing Treatment in Bissell Point WWTP with Three Alternatives for 1 kg Dry Solids Using ReCipe Endpoint Method (H) V1.05/World ReCipe H/A/Weighting.

To improve environmental performance, a comparative analysis was conducted between the existing and the proposed alternative scenarios. Fig. 3 shows the comparison after the normalization and weighting. The Figure emphasizes that most impacts are related to the categories of fossil fuel depletion, human toxicity, particulate matter formation, and climate change.

The fluid bed incineration has beneficial impacts on categories of human toxicity, while the anaerobic digestion scenarios have beneficial impacts on climate change and fossil fuel depletion. The fluid bed incineration shows the beneficial environmental impacts on human toxicity due to recovering energy from sludge combustion and due to lower emissions from this type of incinerator. The design of the fluidized bed incinerator allows complete combustion to be achieved with 20–50% excess air. This represents half of the excess air required by a multiple hearths incinerator and resulted in lower fuel consumption (USDOT, 2012). Also, the detention time for gases in a fluid bed incinerator is typically longer than in a multiple hearths incinerator (6 to 8 s compare to 1 to 2 s). This allows more combustion and less emission, which resulted in lower impacts (Dangtran et al., 2000). The hydrocarbon emissions from a fluid bed incinerator are minimal because the exhaust gases are exposed to a temperature of around 760 °C (1400 °F), which removes the need for using an afterburner (USEPA, 1979). Betzler et al. (1996) showed that replacing the multiple hearths incinerator with a fluid bed incinerator results in a reduction of NO_x_ emissions by 96%. This is the reason why the impact on photochemical oxidant formation in the fluid bed incineration scenario is lower than in the multiple hearths incineration scenario (saving 2.37 × 10^−11^ DALY vs. causing 1.16 × 10^−10^ DALY, respectively) (Table 1). Results showed that in the human toxicity category, the fluid bed incineration scenario achieved better performance and was able to save 3.57 × 10^−7^ DALY while the existing scenario cause 2.16 × 10^−7^ DALY per every kg of dry sludge treated in the plant (Table 1).

Anaerobic digestion scenarios in this case study showed better performance in terms of fossil fuel and metal depletion over other scenarios. Parker et al. (1994) showed that anaerobic digestion was able to remove more than 90% of selected organic toxic compounds. Hospido et al. (2010) showed that the digestion of the sludge, regardless of the operational conditions, was able to achieve up to one third reduction in human and terrestrial toxicity impacts. Gonzalez-Gil et al. (2016) showed that the ability of anaerobic digestion to reduce organic micropollutants was not dependent on operational parameters but compound-specific (some organic micropollutats were highly bio-transformed while others were only slightly affected).

The proposed anaerobic digestion scenario in this case study showed relatively high beneficial environmental impacts on climate change mainly due to the generation of electricity from biogas. While the existing scenario results in a loss of 1.06 × 10^−6^ DALY of human life per every dry kg of sludge incinerated in the plant related to climate change, the anaerobic digestion with landfill scenario is able to reduce the loss to 1.18 × 10^−7^ DALY (Table 1). The proposed anaerobic digestion scenario also shows some beneficial environmental impacts on radiation, ozone layer depletion, land use, and minerals due to the utilization of biogas to produce electricity (Table 1). These results are consistent with other studies such as Hospido et al. (2010). Hospido et al. showed that the production of electricity dominates the performance of anaerobic digestion on the global warming category.

Table 1 shows that there is less impact on human toxicity and particulate matter formation from fluid bed incineration than the other scenarios. The anaerobic digestion scenarios with land application have the lowest impact on climate change (both human health and ecosystems). Although the anaerobic digestion releases more methane, the incineration releases more nitrous oxides which has high global warming potential (1CO_2_ = 28 CH_4_ = 265 N_2_O for GWP100 (IPCC, 2014)) (Daelman et al., 2013).

Due to the expected presence of heavy metals in the sludge, the impacts from anaerobic digestion with land application scenario exceeded all other alternatives. Most of the impact from this scenario is related to human toxicity (7.13 × 10^−7^ DALY vs. 1.42 × 10^−8^ DALY and −3.57 × 10^−7^ DALY from anaerobic digestion/landfill and fluid bed incineration scenarios, respectively). Landfilling the dried digestate from the proposed anaerobic digestion scenario has lower environmental impacts than land application. The impacts due to introducing heavy metals to the agricultural land surpass the benefits of avoided fertilizer in this scenario. However, the heavy metal concentration that was used is not from the case study, but from a different WWTP on a different continent (Hospido et al., 2004 and 2005), and the results may change if data were available from the plant.

**Fig. 4:**
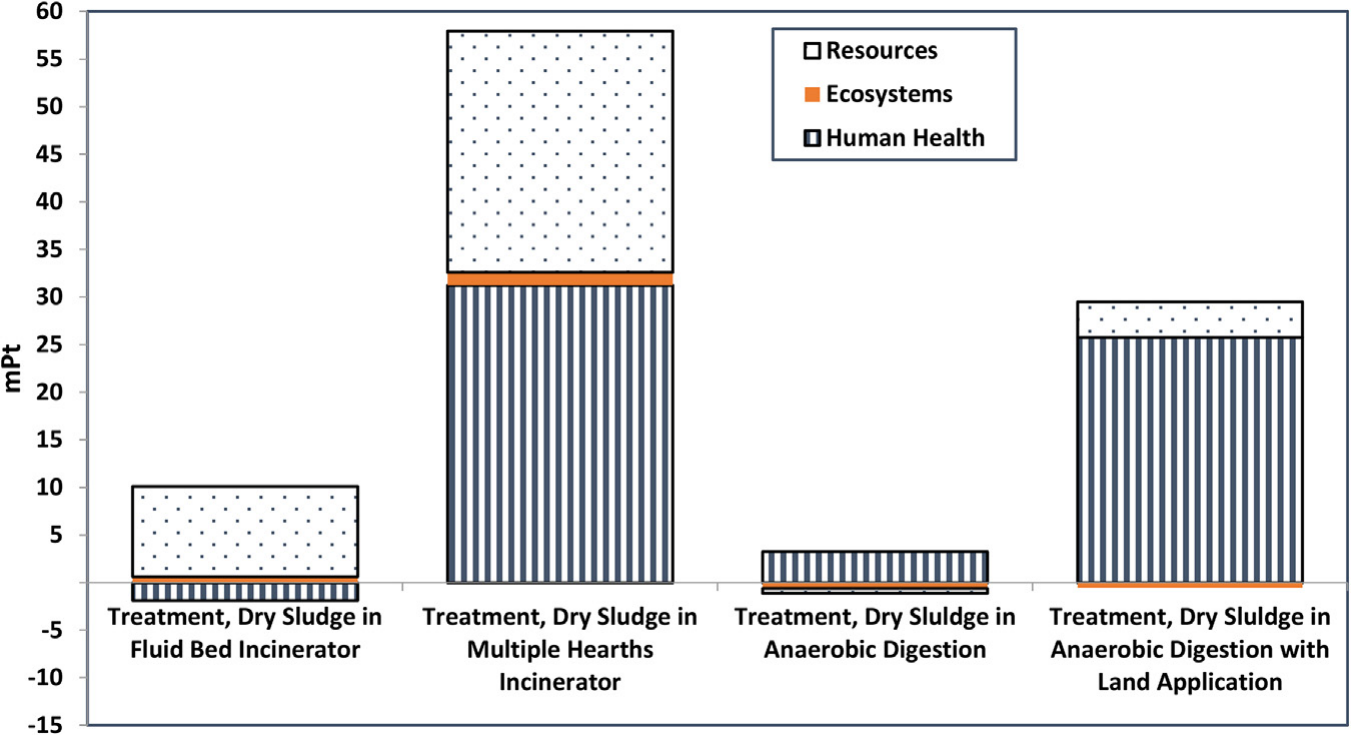
Comparison of Damage Categories of the Existing Treatment in Bissell Point WWTP with Three Alternatives for 1 kg Dry Solids Using ReCipe Endpoint Method (H) V1.05/World ReCipe H/A/Single Score.

On the level of damage categories (human health, ecosystem quality and resources depletion), and as can be seen in Fig. 4 and Table 1, the damages from multiple hearths incineration are the highest in all categories, while the damages from anaerobic digestion with landfilling are the lowest.

Anaerobic digestion has a beneficial impact on resources and ecosystem quality, while the incineration in fluid bed has a beneficial impact on human health. Anaerobic digestion had a higher impact on human health than the fluid bed incineration scenario, while the fluid bed incineration has higher impact on resources than anaerobic digestion. Suh and Rousseaux (2002) found that the anaerobic digestion combined with agricultural land application has better performance than the incineration followed by landfilling due to less emissions and energy consumption. The Suh and Rousseaux study assumed no recovery of energy from the incineration processes and different concentrations of heavy metals for land application which may explain the difference of outcomes with this study.

The anaerobic digestion/landfill scenario has the lowest impact calculated in single scores while the existing incineration in multiple hearths has the highest (Table 1). The single score impact in anaerobic digestion is mostly related to particulate matter formation and human toxicity. The greatest contribution to the single score in the fluid bed incineration is caused by fossil fuel use and the effects of climate change on human health.

Table 1 shows that the scenario of fluid bed incineration will have better reduction of impacts than the scenario of anaerobic digestion on human health, while the anaerobic digestion scenario will have savings on resources. Converting the current treatment train into fluid bed incineration may save 43.2 years of humans’ life, save 0.059 species, and make 62% reduction in the expenses needed by future generations to extract resources annually. While converting to anaerobic digestion may save 36.1 years of human’s life, save 0.157 species, and make 101% reduction in the expenses needed by future generations to extract resources annually.

If the decision makers have more concerns about human health and ecosystem quality in the region, they are recommended to convert to fluid bed incineration, while they should convert to anaerobic digestion if they are concerned more about a resources issue. If their concern is not specific, and is about the sustainability in general, the anaerobic digestion would be recommended. In terms of single score, comparative uncertainty analysis showed that there is a 55.4% probability to have lower damages from anaerobic digestion/landfilling treatment than the fluid bed incineration treatment.

Additional parameters related to other processes such as technology used for fly ash treatment, residue stabilization and transportation may affect the environmental balance. Also, this study did not consider the maintenance needed by each scenario, which if considered may affect the results.

## 4 Conclusions

Energy consumption and direct emissions from processes turned out to be the main contributors to the performance of the sludge treatment unit in the WWTPs that use incineration and they decided the preferable scenario. The study shows how it is important to use the sludge as a resource for energy to offset the other burdens related to treatment. Analysis of incineration of sludge in a multiple hearths incinerator showed that most damages are related to human health and depletion in resources.

In this case study, for the purpose of helping decision makers in selecting a treatment train that can lower annual damages from the sludge handling unit in WWTP using endpoint approach, if the plant continues with the existing treatment method, they will cause 40.6 (±62%) DALYs in human life, 0.11 (±88%) species loss, and $1.08 × 10^+8^ (±69%) more expenses on future generations. Scenarios such as fluid bed incineration/landfilling and anaerobic digestion/landfilling can achieve better environmental performance.

The fluid bed incineration scenario causes lower human toxicity, while the anaerobic digestion scenario causes less impact on climate change and fossil fuel depletion. In general, results showed less damage when implementing fluid bed incineration on human health, while there is less damage on ecosystem quality and resources depletion when implementing anaerobic digestion. If the decision makers are not focused on a specific concern in this case study, they are recommended to convert to anaerobic digestion. They are also recommended to test the level of heavy metals in the sludge for possible land application in the future.

Based on the single scores calculations in the ReCipe methodology, the environmental impact order is: multiple hearths incineration > fluid bed incineration > anaerobic digestion. The energy recovery from anaerobic digestion is more than the recovery from fluid bed incineration, and the lower emissions related to incineration and ashes or sludge taken to landfill did not offset the burdens related to energy.

## Declarations

### Author contribution statement

Isam Alyaseri: Conceived and designed the experiments; Performed the experiments; Analyzed and interpreted the data; Contributed reagents, materials, analysis tools or data; Wrote the paper.

Jianpeng Zhou: Conceived and designed the experiments; Analyzed and interpreted the data.

### Funding statement

This research did not receive any specific grant from funding agencies in the public, commercial, or not-for-profit sectors.

### Competing interest statement

The authors declare no conflict of interest.

### Additional information

No additional information is available for this paper.

## Figures and Tables

**Table 1:** Comparison Analysis of Existing and Three Alternative Scenarios in Solids Treatment Unit in BPWWTP in the Impact Assessment, Damage Assessment, and Single Score Levels Using ReCipe Method (CI: 95%).

Category Impact category	Unit	Treatment in multiple hearths incinerator	Treatment in fluid bed incinerator	Treatment in anaerobic digestion/landfill	Treatment in anaerobic digestion/land application
Agricultural land occupation	Species.yr	4.2 × 10^−11^	-2.93 × 10^−11^	-1.84 × 10^−12^	1.05 × 10^−10^
		(±4.25 × 10^−11^)	(±4.70 × 10^−11^)	(±4.59 × 10^−12^)	(±7.70 × 10^−11^)
Climate change ecosystems	Species.yr	2.69 × 10^−9^	1.38 × 10^−9^	-1.21 × 10^−9^	-1.77 × 10^−9^
		(±2.42 × 10^−9^)	(±2.94 × 10^−9^)	(±2.18 × 10^−9^)	(±2.38 × 10^−9^)
Climate change human health	DALY	4.75 × 10^−7^	2.43 × 10^−7^	-2.14 × 10^−7^	-3.13 × 10^−7^
		(±4.27 × 10^−7^)	(±5.19 × 10^−7^)	(±3.86 × 10^−7^)	(±4.21 × 10^−7^)
Fossil depletion	$	2.83 (±1.94)	1.07 (±1.83)	-0.04 (±0.53)	0.41 (±0.59)
Freshwater eco-toxicity	Species.yr	1.55 × 10^−12^	-8.82 × 10^−13^	-9.48 × 10^−14^	-2.28 × 10^−14^
		(±1.02 × 10^−12^)	(±1.48 × 10^−12^)	(±1.19 × 10^−13^)	(±1.19 × 10^−13^)
Freshwater eutrophication	Species.yr	8.73 × 10^−12^	-3.19 × 10^−11^	1.09 × 10^−11^	-9.20 × 10^−13^
		(±1.09 × 10^−11^)	(±4.99 × 10^−11^)	(±3.87 × 10^−12^)	(±8.27 × 10^−13^)
Human toxicity	DALY	2.16 × 10^−7^	-3.57 × 10^−7^	1.42 × 10^−8^	7.13 × 10^−7^
		(±1.21 × 10^−7^)	(±5.46 × 10^−7^)	(±2.16 × 10^−8^)	(±2.02 × 10^−8^)
Ionizing radiation	DALY	1.41 × 10^−9^	-7.82 × 10^−10^	-1.30 × 10^−9^	-8.96 × 10^−10^
		(±1.45 × 10^−9^)	(±1.70 × 10^−9^)	(±1.64 × 10^−9^)	(±1.53 × 10^−9^)
Marine eco-toxicity	Species.yr	3.68 × 10^−15^	-2.66 × 10^−15^	-3.36 × 10^−16^	-1.57 × 10^−16^
		(±2.12 × 10^−15^)	(±4.65 × 10^−15^)	(±3.74 × 10^−16^)	(±3.77 × 10^−16^)
Metal depletion	$	5.88 × 10^−4^	4.80 × 10^−4^	-6.09 × 10^−4^	-5.6 × 10^−4^
		(±1.59 × 10^−4^)	(±1.02 × 10^−3^)	(±5.25 × 10^−4^)	(±5.52 × 10^−4^)
Natural land transformation	Species.yr	5.26 × 10^−12^	9.73 × 10^−12^	-1.08 × 10^−10^	-7.42 × 10^−12^
		(±2.97 × 10^−11^)	(±1.46 × 10^−11^)	(±4.26 × 10^−11^)	(±6.69 × 10^−11^)
Ozone depletion	DALY	1.89 × 10^−11^	-7.92 × 10^−11^	-4.82 × 10^−11^	4.27 × 10^−10^
		(±1.61 × 10^−11^)	(±1.15 × 10^−10^)	(±3.40 × 10^−11^)	(±3.46 × 10^−11^)
Particulate matter formation	DALY	3.65 × 10^−7^	4.86 × 10^−8^	3.19 × 10^−7^	4.60 × 10^−7^
		(±1.59 × 10^−7^)	(±2.26 × 10^−10^)	(±1.01 × 10^−7^)	(±1.03 × 10^−7^)
Photochemical oxidant formation	DALY	1.16 × 10^−10^	-2.37 × 10^−11^	2.69 × 10^−11^	4.46 × 10^−11^
		(±3.81 × 10^−11^)	(±1.07 × 10^−10^)	(±1.94 × 10^−11^)	(±2.21 × 10^−11^)
Terrestrial acidification	Species.yr	3.16 × 10^−11^	7.87 × 10^−12^	4.89 × 10^−11^	7.13 × 10^−11^
		(±1.54 × 10^−11^)	(±1.73 × 10^−11^)	(±1.58 × 10^−11^)	(±1.58 × 10^−11^)
Terrestrial eco-toxicity	Species.yr	3.06 × 10^−11^	-3.11 × 10^−12^	-8.71 × 10^−13^	4.23 × 10^−10^
		(±1.41 × 10^−11^)	(±1.17 × 10^−11^)	(±8.78 × 10^−13^)	(±9.23 × 10^−13^)
Urban land occupation	Species.yr	7.43 × 10^−11^	-2.51 × 10^−12^	1.41 × 10^−11^	-1.16 × 10^−11^
		(±4.32 × 10^−11^)	(±5.44 × 10^−11^)	(±2.65 × 10^−11^)	(±2.72 × 10^−11^)
**Damage assessment**					
Human health	DALY	1.06 × 10^−6^	-6.68 × 10^−8^	1.18 × 10^−7^	8.59 × 10^−7^
		(±6.60 × 10^−7^)	(±1.17 × 10^−6^)	(±4.47 × 10^−7^)	(±4.86 × 10^−7^)
Annual damages		40.6 (±62%)	-2.6 (±175%)	4.5 (±379%)	32.9 (±57%)

Ecosystem quality	Species.yr	2.88 × 10^−9^	1.33 × 10^−9^	-1.24 × 10^−9^	-1.19 × 10^−9^
		(±2.56 × 10^−9^)	(±3.04 × 10^−9^)	(±2.22 × 10^−9^)	(±2.42 × 10^−9^)
Annual damages	Species	0.11 (±88%)	0.05 (±229%)	-0.05 (±179%)	-0.05 (±204%)

Resources	$	2.83 (±1.94)	1.07 (±1.83)	-0.04 (±0.53)	0.41 (±0.59)
Annual damages		1.08 × 10^+8^	4.09 × 10^+7^	-1.53 × 10^+6^	1.57 × 10^+7^
		(±69%)	(±170%)	(±121%)	(±148%)
**Single score**					
**Total**	mPt.	58.3 (±36.9)	8.35 (±50.7)	2.52 (±18.4)	28.6 (±20.2)
