# Supplementary material for: Towards better environmental performance of wastewater sludge treatment using endpoint approach in LCA methodology
Source: Heliyon. 2017 Mar 17;3(3):e00268. doi: 10.1016/j.heliyon.2017.e00268 (PMC5358968; doi:10.1016/j.heliyon.2017.e00268)
Supplement: Supplementary Materials [file mmc1.docx]

**LCI for Wastewater Sludge Treatment in Bissell Point WWTP**

**Supplementary Materials**

**Table of Contents**

**Figure S1:** Flow Sheet for Sludge Incineration in the Bissell Point WWTP ………...…………..2

**Table S1:** Inventory Data for Existing Scenario in Bissell Point WWTP……………………......2

**Table S2:** Emissions to Air and Water from the Treatment of 1 Dry Kg of Sludge in Multiple Hearths Incinerator. All Data have Lognormal Distribution…………….……………………5

**Table S3:** Inventory Data for Fluid Bed Incineration Scenario……………….………………….9

**Table S4:** Emissions to Air and Water from the Treatment of 1 Dry Kg of Sludge in Fluid Bed Incinerator. All Data have Lognormal Distribution………………………...………………..11

**Table S5:** Inventory Data for the Anaerobic Digestion Scenario……………………………….15

**Table S6:** Emissions to Air and Water from the Treatment of 1 Dry Kg of Sludge in Anaerobic Digestion. All Data have Lognormal Distribution………………………….………...……..19

**Table S7:** Inventory Data for the Anaerobic Digestion/Land Application Scenario....................21

**Table S8:** Emissions to Air and Water from the Treatment of 1 Dry Kg of Sludge in Anaerobic Digestion/Land Application Scenario………………………………………………………..25

**References**…………………………………………………………………………………….…26

**Fig S1**. Flow Sheet for Sludge Incineration in the Bissell Point WWTP.

**Table S1:** Inventory Data for Existing Scenario in Bissell Point WWTP.

| **Inventory** | **Units/** | **Typical** | **Distribution** | **Uncertainty**^a^ | **Data Source^b^** |
| --- | --- | --- | --- | --- | --- |
| **Inputs** | | | | |  |
| Polymer to BFP | kg/dry ton | 1.51 | Lognormal | 3.31 | Provided from the plant |
| Electricity (entire solids handling unit) | KWh/dry kg | 0.353 | Lognormal | 6.16 | Provided from the plant |
| Land occupied by BFP | m^2^ | 1394 | Normal | 269 | Measured onsite |
| Steel materials for BFP | kg | 125872 | Normal | 10206 | Measured onsite |
| Concrete for stands | kg | 82871 | Normal | 8287 | Measured onsite |
| Steel for stands | kg | 2858 | Normal | 285 | Measured onsite |
| Steel for scum concentrator | kg | 11197 | Normal | 215 | Measured onsite |
| Steel for piping | kg | 9385 | Normal | 905 | Measured onsite |
| Fuel for incinerators (natural gas) | MJ/dry kg | 3.49 | Lognormal | 4.79 | Provided from the plant |
| Land occupied by incinerators | m^2^ | 883 | Normal | 46 | Measured onsite |
| Steel for incinerators. | kg | 1378059 | Normal | 235278 | Measured onsite |
| Brick for stock | kg | 506113 | Normal | 27311 | Measured onsite |
| Aluminum for venting | kg | 11499 | Normal | 2300 | Measured onsite |
| HH Alloy for incinerators | kg | 85284 | Normal | 6560 | Measured onsite |
| Concrete for incinerators | kg | 4933 | Normal | 980 | Measured onsite |
| Concrete for ash slurry holding tank | kg | 108862 | Normal | 4536 | Measured onsite |
| Steel for ash slurry holding tank | kg | 3629 | Normal | 227 | Measured onsite |
| Polyurethane used for slurry ash piping | kg | 16556 | Normal | 675 | Measurement onsite |
| Land occupied by ash slurry pond | m^3^ | 20100 | Normal | 980 | Measured onsite |
| Stones used for constructing the pond | kg | 10061150 | Normal | 453592 | calculated onsite |
| Bricks for pond’s floor | kg | 5755905 | Normal | 131542 | Measured onsite |
| Concrete used for walk way | kg | 479864 | Normal | 22680 | Measured onsite |
| Steel used for walk way | kg | 16547 | Normal | 453 | Measurement onsite |
| Steel used for walk way hand rail | kg | 680 | Normal | 69 | Measurement onsite |
| Steel for piping | kg | 9385 | Normal | 725 | Measurement onsite |
| Steel for pumps | kg | 4890 | Normal | 685 | Measurement onsite |
| Transportation of ash to landfill | kg.km/ dry kg | (0.23 kg dry ash produced per dry kg of solids) * 9.65 km the distance to landfill | | | EPA (1979) **^c^** |
| **Outputs** | | | | |  |
| Washwaterfrom BFP | L/dry kg | 18 | Lognormal | 1.59 | Calculated from data given by plant |
| Heat from incineration | MJ/dry kg | 7.52 | lognormal |  | Calculated based on Stillwell et al., 2010**^d^** |
| Dry ash produced | kg/dry kg | 0.23 | lognormal | 1.46 | EPA (1979) |

**^a^**In a 95% confidence interval, the materials that have normal distributions, the uncertainties reported are the double arithmetic standard deviations. While for the materials that have lognormal distributions, the uncertainties reported are the square geometric standard deviations. The method used for uncertainty calculation was described by Frischknechtand Jungbluth (2007). The calculation of uncertainties for this case study can be found in (Alyaseri, 2014).

**^b^**Data collected from Bissell Point WWTP for construction materials were collected by measurements onsite. Data for electricity, natural gas, dry tons incinerated, heat produced, polymers, and dewatering washwater were provided by the plant for the year 2011.

**^c^**Ash from incineration was calculated based on the criteria provided by EPA (1979) to be 0.23 kg of bottom ash/kg of solids incinerated. This value was consistent with range of 20 to 30% of solids in slurry ash from incineration reported by Metcalf and Eddy (2003). The unit kg.km is the weight of ash in kg multiplied by the distance to the landfill in km. This unit is required by SimaPro to calculate the environmental burdens associated to the transportation.

**^d^**The heat waste from incineration was calculated based on Stillwell et al., (2010) equation:

$$ER=\frac{\left( DFR*CS*HV \right)}{\left( HR*DSB \right)}\ldots\ldots\ldots\ldots\ldots\ldots\ldots\ldots\ldots\ldots\ldots\ldots(6)$$

Where *ER* is the heat generated from incineration (MJ/kg of solids). This heat is released to the atmosphere. *DFR* is the daily flow rate in MGD. *CS* is the dry solids content in the wastewater, kg of solids per million gallon. *HV* is the solids heating value, kJ/kg of solids. *HR* represents the steam electric heat rate, kJ/KWh. DSB represents the daily solids burned in the plant (average of 106 dry ton of solids/day).

The wastewater dry solids content ranges between 680 to 1020 kg per million gallon (Metcalf and Eddy, 2003) and the average in this study was taken as 730 kg/MG. The solids heating value has a wide range depend on the type of sludge. Primary sludge may have a higher heating value of up to 29000 KJ/dry kg (Metcalf and Eddy, 2003) while anaerobically digested sludge has a lower heating value of no more than 7500 KJ/dry kg (Murray et al., 2008). However, because the secondary sludge is returned to the primary clarifiers and mixed with primary sludge, the study considered mixed untreated sludge. The range of heating value was assumed to be 20000 to 23210 KJ/dry kg with average of 21610 KJ/dry kg. The steam electric heat rate was assumed to be 10550 kJ/kWh with a conversion factor of 3.6 MJ/KWh (Stillwell et al., 2010).

**Table S2**: Emissions to Air and Water from the Treatment of 1 Dry kg of Sludge in Multiple Hearths Incinerator. Data are for Incineration Process Only. All Data have Lognormal Distribution.

| **Emission Type** | **Unit** | **Typical** | **Data Source** | **Uncertainty** | **Remarks*** |
| --- | --- | --- | --- | --- | --- |
| **Emissions to Air** | | | | | |
| Sulfur dioxide | kg/D. kg | 6.05E-04 | USEPA**_,_**2011 | 1.11 | (1,1,1,1,1,1,4) |
| Carbon dioxide, biogenic | kg/D. kg | 7.26E-02 | Doka, 2006 | 1.91 | Uncertainty calculated by Doka, 2006 |
| Nitrogen oxides | kg/D. kg | 1.85E-03 | USEPA**_,_**2011 | 1.79 | (1,1,1,1,1,5,4) |
| Ammonia | kg/D. kg | 1.68E-06 | Doka, 2006 | 10.44 | Uncertainty calculated by Doka, 2006 |
| Dinitrogen monoxide | kg/D. kg | 9.79E-06 | Doka, 2006 | 9.83 |  |
| Cyanide | kg/D. kg | 1.91E-06 | Doka, 2006 | 10.44 |  |
| Arsenic | kg/D. kg | 5.45E-07 | USEPA, 1995 | 19.00 | Uncertainty covers the range reported by EPA, 1995 |
| Cadmium | kg/D. kg | 2.73E-06 | USEPA, 1995 | 5.38 | (1,5,4,2,4,5,3) |
| Chromium | kg/D. kg | 9.27E-07 | USEPA, 1995 | 8.83 | Uncertainty covers the range reported by EPA, 1995 |
| Copper | kg/D. kg | 1.82E-06 | USEPA, 1995 | 5.39 | (1,5,4,2,4,5,4) |
| Mercury | kg/D. kg | 4.55E-09 | USEPA, 1995 | 5.38 | (1,5,4,2,4,5,3) |
| Manganese | kg/D. kg | 7.73E-07 | USEPA, 1995 | 1.90 | (1,5,4,2,4,5,4) |
| Nickel | kg/D. kg | 8.18E-07 | USEPA, 1995 | 5.39 | (1,5,4,2,4,5,4) |
| Lead | kg/D. kg | 3.44E-09 | USEPA**_,_**2011 | 5.27 | (1,1,1,1,1,5,4) |
| Tin | kg/D. kg | 7.27E-06 | USEPA, 1995 | 1.90 | (1,5,4,2,4,5,4) |
| Zinc | kg/D. kg | 2.18E-05 | USEPA, 1995 | 5.39 | (1,5,4,2,4,5,4) |
| Silicon | kg/D. kg | 4.00E-05 | USEPA, 1995 | 1.90 | (1,5,4,2,4,5,4) |
| Calcium | kg/D. kg | 2.36E-04 | USEPA, 1995 | 1.90 | (1,5,4,2,4,5,4) |
| Aluminum | kg/D. kg | 3.45E-05 | USEPA, 1995 | 1.90 | (1,5,4,2,4,5,4) |
| Magnesium | kg/D. kg | 3.82E-06 | USEPA, 1995 | 1.90 | (1,5,4,2,4,5,4) |
| Heat, waste | MJ/D. kg | 7.52E+00 | Calculated based on Stillwell et al., 2010 equation | | |
| Particulates | kg/D. kg | 1.09E-03 | USEPA**_,_**2011 | 3.33 | (1,5,4,2,4,5,3) |
| Hydrogen chloride | kg/D. kg | 9.09E-06 | USEPA, 1995 and MDNR, 2012 | 1.89 | (1,5,4,2,4,5,3) |
| Barium | kg/D. kg | 2.91E-06 | USEPA, 1995 | 5.39 | (1,5,4,2,4,5,4) |
| Potassium | kg/D. kg | 6.36E-06 | USEPA, 1995 | 1.90 | (1,5,4,2,4,5,4) |
| Selenium | kg/D. kg | 5.46E-08 | USEPA, 1995 | 1.90 | (1,5,4,2,4,5,4) |
| Titanium | kg/D. kg | 2.83E-06 | USEPA, 1995 | 5.39 | (1,5,4,2,4,5,4) |
| Carbon monoxide | kg/D. kg | 1.11E-02 | USEPA**_,_**2011 | 5.27 | (1,1,1,1,1,5,4) |
| VOC, volatile organic compounds | kg/D. kg | 1.71E-03 | USEPA**_,_**2011 | 2.24 | (1,1,1,1,1,5,4) |
| Furan | kg/D. kg | 1.18E-12 | USEPA, 1995 | 1.90 | (1,5,4,2,4,5,4) |
| Dioxin, 2,3,7,8 Tetrachlorodibenzo-p- | kg/D. kg | 3.36E-12 | USEPA, 1995 | 1.90 | (1,5,4,2,4,5,4) |
| **Emissions to water** | | | | | |
| Ammonium, ion | kg/D. kg | 2.76E-04 | Doka, 2006 | 1.58 | Uncertainty calculated by Doka, 2006 |
| Nitrogen | kg/D. kg | 1.89E-05 | Doka, 2006 | 1.67 |  |
| BOD5, Biological oxygen demand | kg/D. kg | 6.96E-05 | Doka, 2006 | 1.18 |  |
| COD, Chemical oxygen demand | kg/D. kg | 2.34E-04 | Doka, 2006 | 1.12 |  |
| TOC, Total organic carbon | kg/D. kg | 5.47E-05 | Doka, 2006 | 1.14 |  |
| DOC, Dissolved organic carbon | kg/D. kg | 5.47E-05 | Doka, 2006 | 1.17 |  |
| Sulfate | kg/D. kg | 5.07E-04 | Doka, 2006 | 2.25 |  |
| Nitrate | kg/D. kg | 1.28E-03 | Doka, 2006 | 1.57 |  |
| Phosphate | kg/D. kg | 1.44E-05 | Doka, 2006 | 1.58 |  |
| Chloride | kg/D. kg | 5.70E-05 | Doka, 2006 | 1.58 |  |
| **Emissions to Groundwater** | | | | | |
| BOD5, Biological oxygen demand | kg/D. kg | 1.61E-04 | Doka, 2006 | 1.78 | Uncertainty calculated by Doka, 2006 |
| COD, Chemical oxygen demand | kg/D. kg | 4.92E-04 | Doka, 2006 | 1.78 |  |
| TOC, Total organic carbon | kg/D. kg | 1.95E-04 | Doka, 2006 | 1.78 |  |
| DOC, Dissolved organic carbon | kg/D. kg | 1.95E-04 | Doka, 2006 | 1.78 |  |
| Sulfate | kg/D. kg | 2.33E-03 | Doka, 2006 | 2.67 |  |
| Nitrate | kg/D. kg | 7.62E-05 | Doka, 2006 | 3.31 |  |
| Phosphate | kg/D. kg | 1.18E-04 | Doka, 2006 | 74.31 |  |
| Arsenic, ion | kg/D. kg | 5.02E-08 | Doka, 2006 | 13.12 |  |
| Cadmium, ion | kg/D. kg | 6.52E-10 | Doka, 2006 | 230.36 |  |
| Cobalt | kg/D. kg | 3.28E-07 | Doka, 2006 | 10.82 |  |
| Chromium VI | kg/D. kg | 3.00E-07 | Doka, 2006 | 10.86 |  |
| Copper, ion | kg/D. kg | 1.05E-05 | Doka, 2006 | 7.22 |  |
| Mercury | kg/D. kg | 3.38E-09 | Doka, 2006 | 41.16 |  |
| Manganese | kg/D. kg | 1.06E-05 | Doka, 2006 | 8.16 |  |
| Molybdenum | kg/D. kg | 1.83E-07 | Doka, 2006 | 11.98 |  |
| Nickel, ion | kg/D. kg | 1.14E-06 | Doka, 2006 | 9.50 |  |
| Lead | kg/D. kg | 2.57E-07 | Doka, 2006 | 215.69 |  |
| Tin, ion | kg/D. kg | 4.67E-07 | Doka, 2006 | 13.49 |  |
| Zinc, ion | kg/D. kg | 5.51E-07 | Doka, 2006 | 107.71 |  |
| Silicon | kg/D. kg | 1.20E-04 | Doka, 2006 | 95.48 |  |
| Iron, ion | kg/D. kg | 2.92E-03 | Doka, 2006 | 7.59 |  |
| Calcium, ion | kg/D. kg | 2.04E-03 | Doka, 2006 | 3.11 |  |
| Aluminum | kg/D. kg | 5.13E-04 | Doka, 2006 | 3.81 |  |
| Magnesium | kg/D. kg | 2.43E-04 | Doka, 2006 | 4.67 |  |

*All data assumed to have lognormal distribution. The uncertainty represents the square geometric standard deviation that covers 95% of the possible data. The 7 numbers in parentheses refers to the ranking (from 1 to 5) of seven data quality indicators used to estimate uncertainty of the data that are not enough to run a goodness of fit test as described by Frischknecht and Jungbluth, (2007).Emissions to water and groundwater with their uncertainties were taken from Doka (2006).

**Table S3:** Inventory Data for a Fluid Bed Incineration Scenario.

| **Parameter** | **Units** | **Typical** | **Distribution** | **Uncertainty^a^** | **Data Source^b^** |
| --- | --- | --- | --- | --- | --- |
| **Inputs** | | | | |  |
| Polymer to BFP | kg/dry ton | 1.51 | Lognormal | 3.31 | Same as MHI |
| Electricity (entire solids handling unit) | kWh/dry kg | 0.344 | Lognormal | 2.48 | Calculated (criteria from USEPA, 1979) |
| Land Occupied by BFP | m^2^ | 1394 | Normal | 269 | Same as MHI |
| Steel materials for BFP | kg | 125872 | Normal | 10206 | Same as MHI |
| Concrete for stands | kg | 82871 | Normal | 8287 | Same as MHI |
| Steel for stands | kg | 2858 | Normal | 285 | Same as MHI |
| Steel for scum concentrator | kg | 11197 | Normal | 215 | Same as MHI |
| Steel for piping | kg | 9385 | normal | 905 | Same as MHI |
| Auxiliary fuel for FB Incineration | MJ/dry kg | 3.28 | lognormal | 1.71 | USEPA, 1979^c^ (4,4,5,2,4,5,4) |
| Land Occupied by FB Incinerators | m^2^ | 883 | Normal | 46 | Same as MHI |
| Refractory Brick for Incinerator | kg | 26522 | Normal | 6949 | Estimated based on the design |
| Steel for FB Incinerator and heat boiler | kg | 56067 | Normal | 3389 | Estimated based on the design |
| Brick for stock for FB Incinerator | kg | 506113 | Normal | 27311 | Same as MHI |
| Aluminum for FB incinerator (venting) | kg | 11499 | Normal | 2300 | Same as MHI |
| Concrete for FB Incinerator for the flat base | kg | 355162 | Normal | 21503 | Estimated based on the design |
| Concrete for ash slurry holding tank | kg | 108862 | Normal | 4536 | Same as MHI |
| Steel for ash slurry holding tank | kg | 3628 | Normal | 227 | Same as MHI |
| Polyurethane used for slurry ash piping to holding pond | kg | 16556 | Normal | 675 | Same as MHI |
| Land Occupied by ash slurry pond | M^3^ | 20100 | Normal | 980 | Same as MHI |
| Stones used for constructing the pond | kg | 10061150 | Normal | 453592 | Same as MHI |
| Concrete used for walk way | kg | 479864 | Normal | 22680 | Same as MHI |
| Bricks for pond’s floor | kg | 5755905 | Normal | 131542 | Same as MHI |
| Steel used for walk way | kg | 16547 | Normal | 453 | Same as MHI |
| Steel used for walk way hand rail | kg | 680 | Normal | 69 | Same as MHI |
| Steel for piping | kg | 9384 | Normal | 725 | Same as MHI |
| Steel for pumps | kg | 4890 | Normal | 685 | Same as MHI |
| Transportation of ash to landfill | kg.km/dry kg | 0.23 kg of as per dry kg of solids * 9.65 km the distance to landfill | | | USEPA (1979)^d^ |
| **Outputs** | | | | |  |
| Washwater from BFP | L/dry kg | 18 | Normal | 1.59 | Same as MHI |
| WW from treatment of fly ash | L/dry kg | 113 | lognormal | 1.31 | Estimated based on the design |
| Heat from FB Incinerator | MJ/dry kg | 7.52 | lognormal |  | Calculated based on Stillwell et al., 2010 |
| Dry ash produced | kg/dry kg | 0.23 | lognormal | 1.46 | USEPA (1979) |

**^a^**In a 95% confidence interval, the materials that have normal distributions, the uncertainties reported are the double arithmetic standard deviations, while for the materials that have lognormal distributions, the uncertainties reported are the square geometric standard deviations. The method used for uncertainty calculation was described by (Frischknecht and Jungbluth, 2007). The calculation of uncertainties for this case study can be found in (Alyaseri, 2014).

**^b^** Some construction materials were assumed to be the same as the Multiple Hearths Incineration scenario (MHI).

**^c^**The supplemental fuel for the fluid bed incinerator was calculated based on a feed rate of 1806 dry Ib/hour as described by USEPA, 1979.

**^d^**Ash from incineration was calculated based on the criteria provided by USEPA (1979) to be 0.23 kg of bottom ash/kg of solids incinerated. This value was consistent with a range of 20 to 30% of solids in slurry ash from incineration reported by Metcalf and Eddy (2003). The unit kg.km is the weight of ash in kg multiplied by the distance to the landfill in km. This unit is required by SimaPro to calculate the environmental burdens associated to the transportation

**Table S4:** Emissions to Air and Water from the Treatment of 1 Dry kg of Sludge in Fluid Bed Incinerator. Data are for Incineration Process Only. All Data have Lognormal Distribution

| Emission Type | Unit | Typical | Source | Uncertainty | Remarks* |
| --- | --- | --- | --- | --- | --- |
| **Emissions to Air** |  |  |  |  |  |
| Sulfuric acid | kg/D. kg | 5.45E-05 | USEPA, 1995 | 1.89 | (1,5,4,2,4,5,3) |
| Carbon dioxide, biogenic | kg/D. kg | 1.50E+00 | Akwo, 2008 from Hospido et al., 2005 | 1.71 | (4,5,2,5,4,5,4) |
| Nitrogen oxides | kg/D. kg | 1.45E-06 | Lederer and Rechberger, 2010. | 2.88 | Uncertainty covers the range reported by Lederer and Rechberger, 2010 |
| Ammonia | kg/D. kg | 1.68E-06 | Doka, 2006 | 10.44 | Uncertainty calculated by Doka, 2006 |
| Dinitrogen monoxide | kg/D. kg | 9.79E-06 | Doka, 2006 | 9.83 |  |
| Cyanide | kg/D. kg | 1.91E-06 | Doka, 2006 | 10.44 |  |
| Arsenic | kg/D. kg | 1.36E-08 | USEPA, 1995 | 5.33 | (1,5,4,2,3,5,3) |
| Cadmium | kg/D. kg | 5.26E-08 | USEPA, 1995 and White et al., 1999 | 9.50 | Uncertainty covers the range between USEPA, 1995 and White et al., 1999 |
| Chromium | kg/D. kg | 1.32E-08 | USEPA, 1995 and White et al., 1999 | 16.39 |  |
| Copper | kg/D. kg | 2.73E-07 | USEPA, 1995 | 5.39 | (1,5,4,2,4,5,4) |
| Mercury | kg/D. kg | 7.35E-08 | USEPA, 1995 and White et al., 1999 | 5.37 | (1,5,3,2,4,5,3) |
| Manganese | kg/D. kg | 2.73E-07 | USEPA, 1995 | 1.90 | (1,5,4,2,4,5,4) |
| Nickel | kg/D. kg | 1.84E-07 | USEPA, 1995 and White et al., 1999 | 7.99 | Uncertainty covers the range between USEPA, 1995 and White et al., 1999 |
| Lead | kg/D. kg | 2.56E-07 | USEPA, 1995 and White et al., 1999 | 270.56 | Uncertainty covers the range reported by USEPA, 1995 |
| Tin | kg/D. kg | 3.18E-07 | USEPA, 1995 | 1.90 | (1,5,4,2,4,5,4) |
| Zinc | kg/D. kg | 9.09E-07 | USEPA, 1995 | 5.39 | (1,5,4,2,4,5,4) |
| Silicon | kg/D. kg | 2.91E-06 | USEPA, 1995 | 1.90 | (1,5,4,2,4,5,4) |
| Calcium | kg/D. kg | 4.55E-06 | USEPA, 1995 | 1.90 | (1,5,4,2,4,5,4) |
| Aluminum | kg/D. kg | 1.73E-06 | USEPA, 1995 | 1.90 | (1,5,4,2,4,5,4) |
| Magnesium | kg/D. kg | 5.45E-07 | USEPA, 1995 | 1.90 | (1,5,4,2,4,5,4) |
| Heat, waste | MJ/D.kg | 1.33E+00 | Calculated based on Stillwell et al., 2010 equation |  |  |
| Particulates | kg/D. kg | 2.38E-04 | Averaged from USEPA, 1972; USEPA, 1995; White et al., 1999; and Neuman et al., 2012 | 8.69 | Uncertainty calculated to cover 95% of the data collected |
| Hydrogen chloride | kg/D. kg | 4.55E-05 | USEPA, 1995 | 1.89 | (1,5,4,2,4,5,3) |
| Barium | kg/D. kg | 2.18E-07 | USEPA, 1995 | 5.39 | (1,5,4,2,4,5,4) |
| Potassium | kg/D. kg | 5.45E-07 | USEPA, 1995 | 1.90 | (1,5,4,2,4,5,4) |
| Selenium | kg/D. kg | 1.82E-07 | USEPA, 1995 | 1.90 | (1,5,4,2,4,5,4) |
| Titanium | kg/D. kg | 3.64E-07 | USEPA, 1995 | 5.39 | (1,5,4,2,4,5,4) |
| **Emissions to water** |  |  |  |  |  |
| Ammonium, ion | kg/D. kg | 2.76E-04 | Doka, 2006 | 1.58 | Uncertainty calculated by Doka, 2006 |
| Nitrogen | kg/D. kg | 1.89E-05 | Doka, 2006 | 1.67 |  |
| BOD5, Biological oxygen demand | kg/D. kg | 6.96E-05 | Doka, 2006 | 1.18 |  |
| COD, Chemical oxygen demand | kg/D. kg | 2.34E-04 | Doka, 2006 | 1.12 |  |
| TOC, Total organic carbon | kg/D. kg | 5.47E-05 | Doka, 2006 | 1.14 |  |
| DOC, Dissolved organic carbon | kg/D. kg | 5.47E-05 | Doka, 2006 | 1.17 |  |
| Sulfate | kg/D. kg | 5.07E-04 | Doka, 2006 | 2.25 |  |
| Nitrate | kg/D. kg | 1.28E-03 | Doka, 2006 | 1.57 |  |
| Phosphate | kg/D. kg | 1.44E-05 | Doka, 2006 | 1.58 |  |
| Chloride | kg/D. kg | 5.70E-05 | Doka, 2006 | 1.58 |  |
| **Emissions to Groundwater** |  |  |  |  |  |
| BOD5, Biological oxygen demand | kg/D. kg | 1.61E-04 | Doka, 2006 | 1.78 | Uncertainty calculated by Doka, 2006 |
| COD, Chemical oxygen demand | kg/D. kg | 4.92E-04 | Doka, 2006 | 1.78 |  |
| TOC, Total organic carbon | kg/D. kg | 1.95E-04 | Doka, 2006 | 1.78 |  |
| DOC, Dissolved organic carbon | kg/D. kg | 1.95E-04 | Doka, 2006 | 1.78 |  |
| Sulfate | kg/D. kg | 2.33E-03 | Doka, 2006 | 2.67 |  |
| Nitrate | kg/D. kg | 7.62E-05 | Doka, 2006 | 3.31 |  |
| Phosphate | kg/D. kg | 1.18E-04 | Doka, 2006 | 74.31 |  |
| Arsenic | kg/D. kg | 5.02E-08 | Doka, 2006 | 13.12 |  |
| Cadmium | kg/D. kg | 6.52E-10 | Doka, 2006 | 230.36 |  |
| Cobalt | kg/D. kg | 3.28E-07 | Doka, 2006 | 10.82 |  |
| Chromium VI | kg/D. kg | 3.00E-07 | Doka, 2006 | 10.86 |  |
| Copper, ion | kg/D. kg | 1.05E-05 | Doka, 2006 | 7.22 |  |
| Mercury | kg/D. kg | 3.38E-09 | Doka, 2006 | 41.16 |  |
| Manganese | kg/D. kg | 1.06E-05 | Doka, 2006 | 8.16 |  |
| Molybdenum | kg/D. kg | 1.83E-07 | Doka, 2006 | 11.98 |  |
| Nickel, ion | kg/D. kg | 1.14E-06 | Doka, 2006 | 9.49 |  |
| Lead | kg/D. kg | 2.57E-07 | Doka, 2006 | 215.69 |  |
| Tin, ion | kg/D. kg | 4.67E-07 | Doka, 2006 | 13.48 |  |
| Zinc, ion | kg/D. kg | 5.51E-07 | Doka, 2006 | 107.71 |  |
| Silicon | kg/D. kg | 1.20E-04 | Doka, 2006 | 95.48 |  |
| Iron, ion | kg/D. kg | 2.92E-03 | Doka, 2006 | 7.59 |  |
| Calcium, ion | kg/D. kg | 2.04E-03 | Doka, 2006 | 3.11 |  |
| Aluminum | kg/D. kg | 5.13E-04 | Doka, 2006 | 3.81 |  |
| Magnesium | kg/D. kg | 2.43E-04 | Doka, 2006 | 4.67 |  |

* All data assumed to have lognormal distribution. The uncertainty represents the square geometric standard deviation that covers 95% of the possible data. The 7 numbers in parentheses refers to the ranking (from 1 to 5) of seven data quality indicators used to estimate uncertainty of the data that are not enough to run a goodness of fit test as described by Frischknecht and Jungbluth, (2007). Emissions to water and groundwater with their uncertainties were taken from Doka (2006).

**Table S5:** Inventory Data for the Anaerobic Digestion Scenario.

| **Parameter** | **Units** | **Typical** | **Distribution** | **Uncertainty^a^** | **Source^b^** |
| --- | --- | --- | --- | --- | --- |
| **Inputs** | | | | | |
| Electricity (for thickening) | kWh/dry kg | 8.89 x10^-4^ | lognormal | 1.53 | EPA, 1979; Goldstein and Smith, 2002; Stillwell et al., 2010; Smith, 1977; and Burton, 1993^c^ |
| Electricity (for digestion) | kWh/dry kg | 0.16 | lognormal | 1.53 | Stillwell et al., 2010; Menendez, 2012; and Butron, 1993^d^ |
| Electricity (for pumping) | kWh/dry kg | 5.5 x10^-3^ | lognormal | 1.53 | Averaged from Goldstein and Smith, 2002 and Burton, 1993 |
| Electricity (for lighting and building) | kWh/dry kg | 16.55 x10^-3^ | lognormal | 1.71 | Smith, 1977 |
| Land Occupied by anaerobic digesters | m^2^ | 9420 | Normal | 465 | Estimated based on the design |
| Reinforcement steel for digesters | kg | 361792 | Normal | 36103 |  |
| Concrete for digesters | kg | 10491963 | Normal | 1046995 |  |
| Construction materials for gravity thickeners and scum concentrators | Reinforcement Steel (kg) | 22125 | Normal | 2177 |  |
|  | Steel for skimmers and bridges (kg) | 8066 | Normal | 807 |  |
|  | Concrete (kg) | 641621 | Normal | 64410 |  |
|  | Steel for scum concentrator (kg) | 11197 | Normal | 215 | Same as MHI |
|  | Steel for piping (kg) | 9385 | normal | 905 | Same as MHI |
| Fuel to heat digester | MJ/dry kg | 0.17 | lognormal | 1.71 | Akwo, 2008 |
| Land Occupied by thickeners | m^2^ | 502 | Normal | 50 | Estimated based on the design |
| Concrete for ash slurry holding tank | kg | 108862 | Normal | 4536 | Same as MHI |
| Steel for ash slurry holding tank | kg | 3629 | Normal | 227 | Same as MHI |
| Polyurethane used for slurry ash piping to holding pond | kg | 16556 | Normal | 675 | Same as MHI |
| Land Occupied by ash slurry pond | m^3^ | 20100 | Normal | 980 | Same as MHI |
| Stones used for constructing the pond | Kg | 10061150 | Normal | 453592 | Same as MHI |
| Bricks for pond’s floor | kg | 5755905 | Normal | 131542 | Same as MHI |
| Concrete used for walk way | kg | 479864 | Normal | 22680 | Same as MHI |
| Steel used for walk way | kg | 16547 | Normal | 453 | Same as MHI |
| Steel used for walk way hand rail | kg | 680 | Normal | 69 | Same as MHI |
| Steel for piping | kg | 9385 | Normal | 725 | Same as MHI |
| Steel for pumps | kg | 4890 | Normal | 685 | Same as MHI |
| Transportation of ash to landfill | kg.km/dry kg | 0.68 kg of ash per dry kg of solids * 9.65 km the distance to landfill=6.54 | | | USEPA, 1985; Tarantini et al., 2007; Akwo, 2008; and Murray et al., 2008**^e^** |
| **Outputs** | | | | |  |
| Energy from biogas produced | KWh/dry kg | 1.94 | lognormal | 2.51 | Stillwell et al., 2010; USDOE, 1981; Burton, 1993; Poulsen and Hansen, 2003; ERG and RDC, 2011; EPA, 1978; Murray et al., 2008; and Johnson, 2006**^f^** |
| Solids to landfill or land application | kg/kg | 0.68 | lognormal | 1.30 | USEPA, 1985; Tarantini et al., 2007; Akwo, 2008; and Murray et al., 2008 |
| Heat loss from digesters | KWh/dry kg | 0.13 | lognormal | 1.65 | Average from ERG and RDG, 2011 and Ghazy et al., 2011 |

**^a^** In a 95% confidence interval, the materials that have normal distributions, the uncertainties reported are the double arithmetic standard deviations.While for the materials that have lognormal distributions, the uncertainties reported are the square geometric standard deviations. The method used for uncertainty calculation was described by (Frischknecht and Jungbluth, 2007). The calculation of uncertainties for this case study can be found in (Alyaseri, 2014).

**^b^** Some construction materials were assumed to be the same as the Multiple Hearths Incineration scenario (MHI).

**^C^** Based on the designed surface area and the EPA (1979) study, the estimated power needed for thickening was 0.19 KWh/dry ton. Goldstein and Smith (2002) reported a power need of 3.4 KWh/dry ton for a 10 MGD plant. Stillwell et al. (2010) reported a power need of 1.90, 2.07, 2.55 and 3.44 KWh/dry ton for a 100, 50, 20 and 10 MGD plant respectively. Smith (1977) reported the amount of this power needed as 13.90, 2.79 and 0.56 KWh/dry ton for a plants have an average flow rates of 1, 10 and 100 MGD, respectively. Burton (1993) reported a 4.13, 3.44, 2.55, 2.07 and 1.86 KWh/dry ton for a plant of 5, 10, 20, 50 and 100 MGD, respectively. A correlation analysis between the power needed and plant size was performed and for the flow rate in the plant, the power needed for thickening was estimated 8.89x10^-4^ KWh/dry kg.

**^d^** Stillwell et al. (2010) estimated the energy requirement as 11,000 KWh/day for a plant of 100 MGD which is equal to 0.152 KWh/dry kg. Menendez (2012) estimated the power needed to be 11% of the entire plant consumption of power. This percentage was applied to the specific data from BPWWTP, and the result was 0.177 KWh/dry kg. Butron (1993) reported that the power needed for AD in a 100 MGD plant using trickling filter was 0.152 KWh/ dry kg. The average of the previous data is 0.16 KWh/dry kg with a lognormal distribution and uncertainty of 1.53.

**^e^**The influent volatile solids is 70% while the volatile solids destroyed through the digestion process is 40% to 60% (EPA, 1985) or 46% (Tarantini et al., 2007). If 46% is assumed to be destroyed, then the solids destroyed 0.7 * 0.46= 32%, and the solids to landfill is 0.68 kg/dry kg digested. This value is close to values reported by Akwo (2008) (0.78 kg/dry kg) and Murray et al. (2008) (0.71 kg/dry kg). The unit kg.km is the weight of ash in kg multiplied by the distance to the landfill in km. This unit is required by SimaPro to calculate the environmental burdens associated to the transportation.

**^f^** Based on Stillwell et al. (2010) equation, a 0.626 KWh will be recovered from each dry kg of total solids went into anaerobic digesters. USDOE (1981) showed that unit methane production from anaerobic digester is not related to the treatment method. The study also shows that the actual production of biogas is not likely expected by calculations and has no effect of region and flow rate on the biogas production. From USDOE (1981) study, the data from 13 plants using trickling filter ranged from 1.54 to 4.96 Kwh/dry kg (CH_4_ is 65% of biogas and the density of CH_4_ was 0.06242796 1b/ft^3^ and the amount of dry tons of sludge per million gallons was 0.73 DT/MG; the net KWh for methane was 14.10 KWh/dry kg; 1 kg of CH_4_ is equal to 55.58 MJ or 15.439 KWh and net heating value for CH_4_ is 910 BTU/ft^3^ (BBBP, 2009)). Energy recovered can be estimated from Burton (1993) to be 0.386 KWh/dry kg. Poulsen and Hansen (2003) reported a production of 734.16 m^3^ for each 1 dry ton of sludge digested, and this value can be changed to 3.76 KWh/dry kg. ERG and RDC (2011) reported a biogas production of 10,000 ft^3^/MGD. This value can be changed to 2.599 KWh/dry kg. The study reported that electricity can be produced as equal to 0.842 KWh/dry kg. EPA (1978) reported that 390 m^3^ can be generated from each dry ton sludge which can be changed to 2.612 KWh/dry kg. Murray et al. (2008) reported that a production of CH_4_ was 227.5 kg/dry ton and the value can be changed to 3.207 KWh/dry kg. Johnson (2006) conducted a survey on several plants and estimated the amount of energy recovered is ranging from 1.526 to 4.880 KWh/dry kg. The data from all previous studies ranged from as low as 0.386 KWh/dry kg (Burton, 1993) to as high as 4.96 KWh/dry kg (USDOE, 1981). A geometric mean of 1.94 KWh/dry kg was taken as the best guess value with an uncertainty of 2.51 to cover this range.

**Table S6:** Emissions to Air and Water from the Treatment of 1 Dry kg of Sludge in Anaerobic Digestion. All Data have Lognormal Distribution

| Emission Type | Unit | Typical | Source | Uncertainty | Remarks^a^ |
| --- | --- | --- | --- | --- | --- |
| **Emissions to Air** | | | | | |
| Heat, waste | kWh/D. kg | 6.56E-03 | Ghazy et al., 2011 | 1.66 | (4,4,2,5,3,5,4) |
| Methane | kg/D. kg | 5.50E-03 | Akwo, 2008 | 1.56 | (2,4,2,5,3,5,4) |
| Carbon dioxide, biogenic | kg/D. kg | 1.29E+00 | Hospido et al., 2005 | 1.56 | (2,4,2,5,3,5,4) |
| Carbon monoxide | kg/D. kg | 8.40E-04 | Hospido et al., 2005 | 1.56 | (2,4,2,5,3,5,4) |
| Nitrogen dioxide | kg/D. kg | 8.50E-04 | Hospido et al., 2005 | 1.75 | (2,4,2,5,3,5,4) |
| Nitric oxide | kg/D. kg | 2.00E-05 | Hospido et al., 2005 | 1.75 | (2,4,2,5,3,5,4) |
| Particulates | kg/D. kg | 8.00E-05 | Hospido et al., 2005 | 3.27 |  |
| NMVOC, non-methane volatile organic compounds | kg/D. kg | 1.38E-05 | Hospido et al., 2010 | 2.28 | (3,4,2,5,3,5,4) |
| Ammonia | kg/D. kg | 3.38E-03 | Hospido et al., 2010 | 1.83 | (3,4,2,5,3,5,4) |
| **Emissions to Water** | | | | | |
| Potassium | kg/D. kg | 1.50E-03 | Hospido et al., 2005 | 1.83 | (3,4,2,5,3,5,4) |
| Aluminum | kg/D. kg | 1.00E-03 | Hospido et al., 2005 | 1.83 | (3,4,2,5,3,5,4) |
| Magnesium | kg/D. kg | 3.00E-03 | Hospido et al., 2005 | 1.83 | (3,4,2,5,3,5,4) |
| Phosphate | kg/D. kg | 8.28E-04 | Hospido et al., 2010 | 1.83 | (3,4,2,5,3,5,4) |
| Copper | kg/D. kg | 1.54E-04 | USEPA, 1978 | 1.83 | (3,4,2,5,3,5,4) |
| Nickel | kg/D. kg | 3.68E-04 | USEPA, 1978 | 1.83 | (3,4,2,5,3,5,4) |
| Chromium | kg/D. kg | 8.82E-04 | USEPA, 1978 | 1.83 | (3,4,2,5,3,5,4) |
| Iron | kg/D. kg | 1.31E-02 | Hospido et al., 2005 | 1.83 | (3,4,2,5,3,5,4) |
| Zinc | kg/D. kg | 7.07E-03 | USEPA, 1978 | 1.83 | (3,4,2,5,3,5,4) |
| Lead | kg/D. kg | 1.50E-03 | USEPA, 1978 | 1.83 | (3,4,2,5,3,5,4) |
| Cadmium | kg/D. kg | 1.37E-04 | USEPA, 1978 | 1.83 | (3,4,2,5,3,5,4) |
| Mercury | kg/D. kg | 5.00E-07 | USEPA, 1978 | 1.83 | (3,4,2,5,3,5,4) |
| **Emissions to Groundwater** | | | | | |
| Sulfate | kg/D. kg | 2.33E-03 | Doka, 2006 | 2.66 | Uncertainty calculated by Doka, 2006 |
| Nitrate | kg/D. kg | 7.62E-05 | Doka, 2006 | 3.31 |  |
| Phosphate | kg/D. kg | 1.18E-04 | Doka, 2006 | 74.31 |  |
| Arsenic | kg/D. kg | 5.02E-08 | Doka, 2006 | 13.12 |  |
| Cadmium | kg/D. kg | 6.52E-10 | Doka, 2006 | 230.36 |  |
| Cobalt | kg/D. kg | 3.28E-07 | Doka, 2006 | 10.82 |  |
| Chromium VI | kg/D. kg | 3.00E-07 | Doka, 2006 | 10.86 |  |
| Copper, ion | kg/D. kg | 1.05E-05 | Doka, 2006 | 7.22 |  |
| Manganese | kg/D. kg | 1.06E-05 | Doka, 2006 | 8.16 |  |
| Molybdenum | kg/D. kg | 2.33E-03 | Doka, 2006 | 11.98 |  |
| Tin, ion | kg/D. kg | 7.62E-05 | Doka, 2006 | 13.49 |  |
| Silicon | kg/D. kg | 1.20E-04 | Doka, 2006 | 95.48 |  |
| Iron, ion | kg/D. kg | 2.92E-03 | Doka, 2006 | 7.59 |  |
| Calcium, ion | kg/D. kg | 2.04E-03 | Doka, 2006 | 3.11 |  |

^a^In a 95% confidence interval, the materials that have normal distributions, the uncertainties reported are the double arithmetic standard deviations.While for the materials that have lognormal distributions, the uncertainties reported are the square geometric standard deviations. The method used for uncertainty calculation was described by (Frischknecht and Jungbluth, 2007). The calculation of uncertainties for this case study can be found in (Alyaseri, 2014).

**Table S7:** Inventory Data for the Anaerobic Digestion/Land Application Scenario.

| **Parameter** | **Units** | **Typical** | **Distribution** | | **Uncertainty^a^** | | **Source^b^** |
| --- | --- | --- | --- | --- | --- | --- | --- |
| **Inputs** | | | | | | | |
| Electricity (for thickening) | kWh/dry kg | 8.89 x10^-4^ | lognormal | | 1.53 | | EPA, 1979; Goldstein and Smith, 2002; Stillwell et al., 2010; Smith, 1977; and Burton, 1993^c^ |
| Electricity (for digestion) | kWh/dry kg | 0.16 | lognormal | | 1.53 | | Stillwell et al., 2010; Menendez, 2012; and Butron, 1993^d^ |
| Electricity (for pumping) | kWh/dry kg | 5.5 x10^-3^ | lognormal | | 1.53 | | Averaged from Goldstein and Smith, 2002 and Burton, 1993 |
| Electricity (for lighting and building) | kWh/dry kg | 16.55 x10^-3^ | lognormal | | 1.71 | | Smith, 1977 |
| Electricity consumption for land application | MJ/dry kg | 0.21 | lognormal | | 1.83 | | Hospido et al., 2005 |
| Land Occupied by anaerobic digesters | m^2^ | 9420 | Normal | | 465 | | Estimated based on the design |
| Reinforcement steel for digesters | kg | 361792 | Normal | | 36103 | |  |
| Concrete for digesters | kg | 10491963 | Normal | | 1046995 | |  |
| Construction materials for gravity thickeners and scum concentrators | Reinforcement Steel (kg) | 22125 | Normal | | 2177 | |  |
|  | Steel for skimmers and bridges (kg) | 8066 | Normal | | 807 | |  |
|  | Concrete (kg) | 641621 | Normal | | 64410 | |  |
|  | Steel for scum concentrator (kg) | 11197 | Normal | | 215 | | Same as MHI |
|  | Steel for piping (kg) | 9385 | normal | | 905 | | Same as MHI |
| Fuel to heat digester | MJ/dry kg | 0.17 | lognormal | | 1.71 | | Akwo, 2008 |
| Land Occupied by thickeners | m^2^ | 502 | Normal | | 50 | | Estimated based on the design |
| Concrete for ash slurry holding tank | kg | 108862 | Normal | | 4536 | | Same as MHI |
| Steel for ash slurry holding tank | kg | 3629 | Normal | | 227 | | Same as MHI |
| Polyurethane used for slurry ash piping to holding pond | kg | 16556 | Normal | | 675 | | Same as MHI |
| Land Occupied by ash slurry pond | m^3^ | 20100 | Normal | | 980 | | Same as MHI |
| Stones used for constructing the pond | kg | 10061150 | Normal | | 453592 | | Same as MHI |
| Bricks for pond’s floor | kg | 5755905 | Normal | | 131542 | | Same as MHI |
| Concrete used for walk way | kg | 479864 | Normal | | 22680 | | Same as MHI |
| Steel used for walk way | kg | 16547 | Normal | | 453 | | Same as MHI |
| Steel used for walk way hand rail | kg | 680 | Normal | | 69 | | Same as MHI |
| Steel for piping | kg | 9385 | Normal | | 725 | | Same as MHI |
| Steel for pumps | kg | 4890 | Normal | | 685 | | Same as MHI |
| Transportation of ash to farms | kg.km/dry kg | 0.68 kg of ash per dry kg of solids * 41.7 km (=25 mile) the distance to farms=28.36 | | | | | USEPA, 1985; Tarantini et al., 2007; Akwo, 2008; and Murray et al., 2008**^e^** |
| lime | kg | 0.2 | |  | |  | Houillon & Jolliet (2005) |
| Diesel for sludge application | kg | 0.00073 | |  | |  | Hospido et al (2005) |
| polymer | kg | 0.0071 | |  | |  | Houillon& Jolliet (2005) |
| Oil needed | kg | 0.03 | |  | |  | Lundin et al, 2004 |
| Natural gas needed | kg | 0.013 | |  | |  | Lundin et al, 2004 |
| **Outputs** | | | | | | |  |
| Energy from biogas produced | KWh/dry kg | 1.94 | lognormal | | 2.51 | | Stillwell et al., 2010; USDOE, 1981; Burton, 1993; Poulsen and Hansen, 2003; ERG and RDC, 2011; EPA, 1978; Murray et al., 2008; and Johnson, 2006**^f^** |
| Solids to land application | kg/kg | 0.68 | lognormal | | 1.30 | | USEPA, 1985; Tarantini et al., 2007; Akwo, 2008; and Murray et al., 2008 |
| Heat loss from digesters | KWh/dry kg | 0.13 | lognormal | | 1.65 | | Average from ERG and RDG, 2011 and Ghazy et al., 2011 |
| **Avoided Products** | | | | | | | |
| Fertilizer N | kg | 37.6E-03 |  | |  | | Average from Hospido et al, 2004 and Pasqualino et al, 2009 |
| Fertilizer P | kg | 0.020 |  | |  | | Hospido et al, 2004 |
| Fertilizer K | kg | 0.0024 |  | |  | | Houillon and Jolliet, 2005 |

**^a^** In a 95% confidence interval, the materials that have normal distributions, the uncertainties reported are the double arithmetic standard deviations. While for the materials that have lognormal distributions, the uncertainties reported are the square geometric standard deviations. The method used for uncertainty calculation was described by (Frischknecht and Jungbluth, 2007). The calculation of uncertainties for this case study can be found in (Alyaseri, 2014).

**^b^** Some construction materials were assumed to be the same as the Multiple Hearths Incineration scenario (MHI).

**^C^** Based on the designed surface area and the EPA (1979) study, the estimated power needed for thickening was 0.19 KWh/dry ton. Goldstein and Smith (2002) reported a power need of 3.4 KWh/dry ton for a 10 MGD plant. Stillwell et al. (2010) reported a power need of 1.90, 2.07, 2.55 and 3.44 KWh/dry ton for a 100, 50, 20 and 10 MGD plant respectively. Smith (1977) reported the amount of this power needed as 13.90, 2.79 and 0.56 KWh/dry ton for a plants have an average flow rates of 1, 10 and 100 MGD, respectively. Burton (1993) reported a 4.13, 3.44, 2.55, 2.07 and 1.86 KWh/dry ton for a plant of 5, 10, 20, 50 and 100 MGD, respectively. A correlation analysis between the power needed and plant size was performed and for the flow rate in the plant, the power needed for thickening was estimated 8.89x10^-4^ KWh/dry kg.

**^d^** Stillwell et al. (2010) estimated the energy requirement as 11,000 KWh/day for a plant of 100 MGD which is equal to 0.152 KWh/dry kg. Menendez (2012) estimated the power needed to be 11% of the entire plant consumption of power. This percentage was applied to the specific data from BPWWTP, and the result was 0.177 KWh/dry kg. Butron (1993) reported that the power needed for AD in a 100 MGD plant using trickling filter was 0.152 KWh/ dry kg. The average of the previous data is 0.16 KWh/dry kg with a lognormal distribution and uncertainty of 1.53.

**^e^**The influent volatile solids is 70% while the volatile solids destroyed through the digestion process is 40% to 60% (EPA, 1985) or 46% (Tarantini et al., 2007). If 46% is assumed to be destroyed, then the solids destroyed 0.7 * 0.46= 32%, and the solids to landfill is 0.68 kg/dry kg digested. This value is close to values reported by Akwo (2008) (0.78 kg/dry kg) and Murray et al. (2008) (0.71 kg/dry kg). The unit kg.km is the weight of ash in kg multiplied by the distance to the landfill in km. This unit is required by SimaPro to calculate the environmental burdens associated to the transportation.

**^f^** Based on Stillwell et al. (2010) equation, a 0.626 KWh will be recovered from each dry kg of total solids went into anaerobic digesters. USDOE (1981) showed that unit methane production from anaerobic digester is not related to the treatment method. The study also shows that the actual production of biogas is not likely expected by calculations and has no effect of region and flow rate on the biogas production. From USDOE (1981) study, the data from 13 plants using trickling filter ranged from 1.54 to 4.96 Kwh/dry kg (CH_4_ is 65% of biogas and the density of CH_4_ was 0.06242796 1b/ft^3^ and the amount of dry tons of sludge per million gallons was 0.73 DT/MG; the net KWh for methane was 14.10 KWh/dry kg; 1 kg of CH_4_ is equal to 55.58 MJ or 15.439 KWh and net heating value for CH_4_ is 910 BTU/ft^3^ (BBBP, 2009)). Energy recovered can be estimated from Burton (1993) to be 0.386 KWh/dry kg. Poulsen and Hansen (2003) reported a production of 734.16 m^3^ for each 1 dry ton of sludge digested, and this value can be changed to 3.76 KWh/dry kg. ERG and RDC (2011) reported a biogas production of 10,000 ft^3^/MGD. This value can be changed to 2.599 KWh/dry kg. The study reported that electricity can be produced as equal to 0.842 KWh/dry kg. EPA (1978) reported that 390 m^3^ can be generated from each dry ton sludge which can be changed to 2.612 KWh/dry kg. Murray et al. (2008) reported that a production of CH_4_ was 227.5 kg/dry ton and the value can be changed to 3.207 KWh/dry kg. Johnson (2006) conducted a survey on several plants and estimated the amount of energy recovered is ranging from 1.526 to 4.880 KWh/dry kg. The data from all previous studies ranged from as low as 0.386 KWh/dry kg (Burton, 1993) to as high as 4.96 KWh/dry kg (USDOE, 1981). A geometric mean of 1.94 KWh/dry kg was taken as the best guess value with an uncertainty of 2.51 to cover this range.

**Table S8:** Emissions to Air and Water from the Treatment of 1 Dry kg of Sludge in Anaerobic Digestion/Land Application. All Data have Lognormal Distribution

| **Emission Type** | **Unit** | **Typical** | **Source** | **Uncertainty** | **Remarks^a^** |
| --- | --- | --- | --- | --- | --- |
| **Emissions to Air** | | | | | |
| Heat, waste | kWh/D. kg | 6.56E-03 | Ghazy et al., 2011 | 1.66 | (4,4,2,5,3,5,4) |
| Methane | kg/D. kg | 5.50E-03 | Akwo, 2008 | 1.56 | (2,4,2,5,3,5,4) |
| Carbon dioxide, biogenic | kg/D. kg | 1.29E+00 | Hospido et al., 2005 | 1.56 | (2,4,2,5,3,5,4) |
| Carbon monoxide | kg/D. kg | 8.40E-04 | Hospido et al., 2005 | 1.56 | (2,4,2,5,3,5,4) |
| Nitrogen dioxide | kg/D. kg | 8.50E-04 | Hospido et al., 2005 | 1.75 | (2,4,2,5,3,5,4) |
| Nitric oxide | kg/D. kg | 2.00E-05 | Hospido et al., 2005 | 1.75 | (2,4,2,5,3,5,4) |
| Particulates | kg/D. kg | 8.00E-05 | Hospido et al., 2005 | 3.27 |  |
| NMVOC, non-methane volatile organic compounds | kg/D. kg | 1.38E-05 | Hospido et al., 2010 | 2.28 | (3,4,2,5,3,5,4) |
| CH4 from land application | kg/D. kg | 3.18E-03 | Hospido et al., 2005 | 1.56 | (2,4,2,5,3,5,4) |
| NH3 from land application | kg/D. kg | 1.9E-03 | Svanstrom et al., 2005 | 1.56 | (2,4,2,5,3,5,4) |
| NOx from land application | kg/D. kg | 0.82 E-03 | Svanstrom et al., 2005 | 1.56 | (2,4,2,5,3,5,4) |
| CO_2_ | kg/D. kg | 25E-03 | Lundin et al, 2004 | 1.56 | (2,4,2,5,3,5,4) |
| Ammonia | kg/D. kg | 3.38E-03 | Hospido et al., 2010 | 1.83 | (3,4,2,5,3,5,4) |
| **Emissions to Water** | | | | | |
| Potassium | kg/D. kg | 1.50E-03 | Hospido et al., 2005 | 1.83 | (3,4,2,5,3,5,4) |
| Aluminum | kg/D. kg | 1.00E-03 | Hospido et al., 2005 | 1.83 | (3,4,2,5,3,5,4) |
| Magnesium | kg/D. kg | 3.00E-03 | Hospido et al., 2005 | 1.83 | (3,4,2,5,3,5,4) |
| Phosphate | kg/D. kg | 8.28E-04 | Hospido et al., 2010 | 1.83 | (3,4,2,5,3,5,4) |
| Copper | kg/D. kg | 1.54E-04 | USEPA, 1978 | 1.83 | (3,4,2,5,3,5,4) |
| Nickel | kg/D. kg | 3.68E-04 | USEPA, 1978 | 1.83 | (3,4,2,5,3,5,4) |
| Chromium | kg/D. kg | 8.82E-04 | USEPA, 1978 | 1.83 | (3,4,2,5,3,5,4) |
| Iron | kg/D. kg | 1.31E-02 | Hospido et al., 2005 | 1.83 | (3,4,2,5,3,5,4) |
| Zinc | kg/D. kg | 7.07E-03 | USEPA, 1978 | 1.83 | (3,4,2,5,3,5,4) |
| Lead | kg/D. kg | 1.50E-03 | USEPA, 1978 | 1.83 | (3,4,2,5,3,5,4) |
| Cadmium | kg/D. kg | 1.37E-04 | USEPA, 1978 | 1.83 | (3,4,2,5,3,5,4) |
| Mercury | kg/D. kg | 5.00E-07 | USEPA, 1978 | 1.83 | (3,4,2,5,3,5,4) |
| **Emissions to Soil** | | | | | |
| Cr | kg/D. kg | 8E-05 | Hospido et al., 2005 | 1.83 |  |
| Cu | kg/D. kg | 1.9E-04 | Hospido et al., 2005 | 1.83 |  |
| Pb | kg/D. kg | 3.3E-04 | Hospido et al., 2005 | 1.83 |  |
| Zn | kg/D. kg | 1.51E-03 | Hospido et al., 2005 | 1.83 |  |
| Cd | kg/D. kg | 1.38E-06 | Hospido et al., 2004 | 1.83 |  |
| Hg | kg/D. kg | 1.43E-06 | Hospido et al., 2004 | 1.83 |  |
| Ni | kg/D. kg | 2.92E-05 | Hospido et al., 2004 | 1.83 |  |

^a^In a 95% confidence interval, the materials that have normal distributions, the uncertainties reported are the double arithmetic standard deviations. While for the materials that have lognormal distributions, the uncertainties reported are the square geometric standard deviations. The method used for uncertainty calculation was described by (Frischknecht and Jungbluth, 2007). The calculation of uncertainties for this case study can be found in (Alyaseri, 2014).

References

Akwo, N. S. 2008.“A Life Cycle Assessment of Sewage Sludge Treatment Options.” M.S. Thesis, Department of Development and Planning, Aalborg University, Denmark.

Alyaseri, I. 2014. “Qualitative and Quantitative Procedure for Uncertainty Analysis in Life Cycle Assessment of Wastewater Solids Treatment Processes.” Dissertation, Southern Illinois University Carbondale, Carbondale, IL. ISBN: 9781321297959.

Baltic Biogas Bus Project (BBBP). 2009. “Baltic Biogas Bus.” *About Biogas*,<http://www.balticbiogasbus.eu/web/about-biogas.aspx> (Oct. 15, 2013).

Burton, F. 1993.“Water and Wastewater Industries: Characteristics and DSM Opportunities.” EPRI TR-102015, Project 2662-10, 3046-03, Final Report 1993.

Doka, G. 2006.“Disposal, Raw Sewage Sludge, to Municipal Incineration*.* Available on SimaPro-7 Database.”Data entry by Niels Jungbluth, e-mail: esu-services@ecoinvent.org; Company: ESU; Country: CH.

ERG (Eastern Research Group, Inc.) and RDC (Resource Dynamics Corporation). 2011. “Opportunities for Combined Heat and Power at Wastewater Treatment Facilities: Market Analysis and Lessons from the Field. Prepared for USEPA and Combined Heat and Power Partnership (USEPA &CHP), October 2011. http://www.epa.gov/chp/documents/wwtf_opportunities.pdf.Accessed 18 September 2013.

Frischknecht, R., and Jungbluth, N. (Editors). 2007. “Overview and Methodology, Data v2.0 (2007). Ecoinvent Report No. 1. Dubendorf, December 2007. <http://www.ecoinvent.org/fileadmin/documents/en/01_OverviewAndMethodology.pdf.(September 10, 2012).

Ghazy, M. R., Dockhorn, T., and Dichtl, N. 2011. “Economic and Environmental Assessment of Sewage Sludge Treatment Processes Application in Egypt.”Fifteenth International Water Technology Conference, Alexandria, Egypt, IWTC-15 2011.

Goldstein, R., and Smith, W. 2002. “Water & Sustainability (Volume 4): U.S. Electricity Consumption for Water Supply & Treatment—The Next Half Century.”<http://www.rivernetwork.org/resource-library/water-sustainability-volume-4-us-electricity-consumption-water-supply-and-treatment>(January 16, 2013).

Hospido, A., Carballa, M., Moreira, M., Omil, F., Lema, J., and Feijoo, G. 2010.“Environmental Assessment of Anaerobically Digested Sludge Reuse in Agriculture: Potential Impacts of Emerging Micropollutants.”WaterRes 44(10): 3225-3233.

Hospido, A., Moreira, M., Martin, M., Rigola, M., and Feijoo, G. 2005. “Environmental Evaluation of Different Treatment Processes for Sludge from Urban Wastewater Treatments: Anaerobic Digestion versus Thermal Processes.”Int J LCA 10(5): 336-345.

Hospido, A., Moreira, M T., Fernández-Couto, M., and Feijoo, G. 2004. “Environmental performance of a municipal wastewater treatment plant.” Int J LCA 9(4): 261-271.

Johnson, C. 2006.“A Survey of Municipal Thermophilic Anaerobic Sludge Digesters and Diagnostic Activity Assays.” M.S. Thesis, Marquette University, Milwaukee, Wisconsin.

Lederer, J. and Rechberger, H. 2010.“Comparative Goal-Oriented Assessment of Conventional and Alternative Sewage Sludge Treatment Options.” Waste Manage. 30(6): 1043-1056.

Menendez, M. 2012.“How We Use Energy at Wastewater Plants…and How We Can Use Less.”<http://www.ncsafewater.org/Pics/Training/AnnualConference/AC10TechnicalPapers/AC10_Wastewater/WW_T.AM_10.30_Menendez.pdf>(November 30, 2012).

Metcalf and Eddy, Inc. 2003.“Wastewater Engineering: Treatment and Reuse.” Tata McGraw-Hill, 4^th^ Edition, ISBN-13:978-0-07-049539-5, ISBN-10: 0-07-049539-4.

Missouri Department of Natural Resources (MDNR). 2012. “Section 111(D)/129 State Plan for Sewage Sludge Incinerators in Missouri.” Division of Environmental Quality Air Pollution Control Program, Jefferson City, Missouri, Prepared for the Missouri Air Conservation Commission.<http://www.dnr.mo.gov/env/apcp/public-notices/111d-plan-for-sewage-sludge-incinerators-for-public-notice.pdf>(March 22, 2013).

Murray, A., Horvath, A., and Nelson, K. 2008. “Hybrid Life Cycle Environment and Cost Inventory of Sewage Sludge Treatment and End-Use Scenarios: A Case Study from China.”Env. Sci. & Techno. 42(9): 3163-3169.

Neuman, M., Smith, S., Henry, C., and Moll, T. 2012.“Literature Review on Air Emissions and Ash Resulting from Incineration of Biosolids.” University of Washington College of Forest Resources, Seattle, Washington 98195. <http://faculty.washington.edu/clh/litrev/IncinSum.pdf>(November 28, 2012).

Poulsen, T. and Hansen, J. 2003.“Strategic Environmental Assessment of Alternative Sewage Sludge Management Scenarios.”Waste Management & Research21(1): 19-28.

Smith, J. 1977. “Inventory of Energy Use in Wastewater Sludge Treatment and Disposal.” Indust. Water Eng., 14(4): 20-26.

Stillwell, A., Hoppock, D., and Webber, M. 2010.“Energy Recovery from Wastewater Treatment Plants in the United States: A Case Study of the Energy-Water Nexus.” Sustainability 2010 2(4): 945-962.

Tarantini, M.,Buttol, P., Maiorino, L. 2007.“An Environmental LCA of Alternative Scenarios of Urban Sewage Sludge Treatment and Disposal.”Therm. Sci., 11(3): 153-164.

U. S. DOE. 1981.“Production and Utilization of Methane from Anaerobic Sludge Digestion in U.S. Wastewater Treatment Plants.” DOE/CS/20300-3, distribution UC-95e.

U. S. EPA. 1972.“Sewage Sludge Incineration.” PB 211 323, Washington DC.

U.S. EPA. 1978.“Sludge Treatment and Disposal: Sludge Treatment Volume 1.” EPA-625/4-78-012.

U. S. EPA. 1979.“Process Design Manual Sludge Treatment and Disposal.” EPA-625/1-79-011.

U. S. EPA. 1985. “Handbook, Estimating Sludge Management Costs.” Ed, EPA, 1985 Cincinnati, 540.

U.S. EPA. 1995. “Compilation of Air Pollutant Emission Factors.” AP-42, Volume I, Chapter 2, Sewage Sludge Incineration.<http://www.epa.gov/ttnchie1/ap42/ch02/final/c02s02.pdf>(February 11, 2013).

U.S. EPA. 2011. National Emission Inventory, Base Emissions from Solids Incineration in Bissell Point Wastewater Treatment Plant, St. Louis, MO. Retrieved from Planenthazar.com. <http://www.planethazard.com/phpolluters.aspx?mode=polluters&area=state&state=MO&sort=emissions>.(March 5, 2013).

White, A., Mullen, J., and Mayrose, D. 1999.“Replacement of a Multiple Hearth by a Fluid Bed Incinerator: The Greensboro Experience.” 1999 International Conference on Incineration and Thermal Treatment Technologies, Radisson Twin Towers, Orlando, FL.
